# Nasal host response-based screening for undiagnosed respiratory viruses: a pathogen surveillance and detection study

**DOI:** 10.1016/S2666-5247(22)00296-8

**Published:** 2023-01

**Authors:** Nagarjuna R Cheemarla, Amelia Hanron, Joseph R Fauver, Jason Bishai, Timothy A Watkins, Anderson F Brito, Dejian Zhao, Tara Alpert, Chantal B F Vogels, Albert I Ko, Wade L Schulz, Marie L Landry, Nathan D Grubaugh, David van Dijk, Ellen F Foxman

**Affiliations:** Department of Laboratory Medicine, Department of Immunobiology, Yale School of Public Health, New Haven, CT, USA; Department of Laboratory Medicine, Yale School of Medicine, New Haven, CT, USA; Department of Epidemiology of Microbial Diseases, Yale School of Public Health, New Haven, CT, USA; Department of Pathology and Laboratory Medicine, University of Washington, Seattle, WA, USA; Yale School of Medicine, New Haven, CT, USA; Department of Epidemiology of Microbial Diseases, Yale School of Public Health, New Haven, CT, USA; Department of Epidemiology, University of Nebraska Medical Center, Omaha, NE, USA; Department of Ecology and Evolutionary Biology, Yale University, New Haven, CT, USA; Department of Laboratory Medicine, Department of Immunobiology, Yale School of Public Health, New Haven, CT, USA; Yale School of Medicine, New Haven, CT, USA; Department of Epidemiology of Microbial Diseases, Yale School of Public Health, New Haven, CT, USA, Instituto Todos pela Saúde, São Paulo, Brazil; Department of Genetics, Yale School of Public Health, New Haven, CT, USA; Yale School of Medicine, New Haven, CT, USA; Department of Epidemiology of Microbial Diseases, Yale School of Public Health, New Haven, CT, USA; Yale School of Medicine, New Haven, CT, USA; Department of Epidemiology of Microbial Diseases, Yale School of Public Health, New Haven, CT, USA; Department of Internal Medicine, Yale School of Medicine, New Haven, CT, USA; Department of Epidemiology of Microbial Diseases, Yale Institute of Global Health, Yale School of Public Health, New Haven, CT, USA; Department of Laboratory Medicine, Yale School of Public Health, New Haven, CT, USA; Department of Laboratory Medicine, Yale School of Public Health, New Haven, CT, USA; Yale School of Medicine, New Haven, CT, USA; Department of Epidemiology of Microbial Diseases, Yale Institute of Global Health, Yale School of Public Health, New Haven, CT, USA, Department of Ecology and Evolutionary Biology, Yale University, New Haven, CT, USA; Department of Internal Medicine, Yale School of Public Health, New Haven, CT, USA, Department of Computer Science, Yale University, New Haven, CT, USA; Department of Laboratory Medicine, Department of Immunobiology, Yale School of Public Health, New Haven, CT, USA

## Abstract

**Background:**

Symptomatic patients who test negative for common viruses are an important possible source of unrecognised or emerging pathogens, but metagenomic sequencing of all samples is inefficient because of the low likelihood of finding a pathogen in any given sample. We aimed to determine whether nasopharyngeal CXCL10 screening could be used as a strategy to enrich for samples containing undiagnosed viruses.

**Methods:**

In this pathogen surveillance and detection study, we measured CXCL10 concentrations from nasopharyngeal swabs from patients in the Yale New Haven health-care system, which had been tested at the Yale New Haven Hospital Clinical Virology Laboratory (New Haven, CT, USA). Patients who tested negative for a panel of respiratory viruses using multiplex PCR during Jan 23–29, 2017, or March 3–14, 2020, were included. We performed host and pathogen RNA sequencing (RNA-Seq) and analysis for viral reads on samples with CXCL10 higher than 1 ng/mL or CXCL10 testing and quantitative RT-PCR (RT-qPCR) for SARS-CoV-2. We used RNA-Seq and cytokine profiling to compare the host response to infection in samples that were virus positive (rhinovirus, seasonal coronavirus CoV-NL63, or SARS-CoV-2) and virus negative (controls).

**Findings:**

During Jan 23–29, 2017, 359 samples were tested for ten viruses on the multiplex PCR respiratory virus panel (RVP). 251 (70%) were RVP negative. 60 (24%) of 251 samples had CXCL10 higher than 150 pg/mL and were identified for further analysis. 28 (47%) of 60 CXCL10-high samples were positive for seasonal coronaviruses. 223 (89%) of 251 samples were PCR negative for 15 viruses and, of these, CXCL10-based screening identified 32 (13%) samples for further analysis. Of these 32 samples, eight (25%) with CXCL10 concentrations higher than 1 ng/mL and sufficient RNA were selected for RNA-Seq. Microbial RNA analysis showed the presence of influenza C virus in one sample and revealed RNA reads from bacterial pathobionts in four (50%) of eight samples. Between March 3 and March 14, 2020, 375 (59%) of 641 samples tested negative for 15 viruses on the RVP. 32 (9%) of 375 samples had CXCL10 concentrations ranging from 100 pg/mL to 1000 pg/mL and four of those were positive for SARS-CoV-2. CXCL10 elevation was statistically significant, and a distinguishing feature was found in 28 (8%) of 375 SARS-CoV-2-negative samples versus all four SARS-CoV-2-positive samples (p=4·4 × 10^−5^). Transcriptomic signatures showed an interferon response in virus-positive samples and an additional neutrophil-high hyperinflammatory signature in samples with high amounts of bacterial pathobionts. The CXCL10 cutoff for detecting a virus was 166·5 pg/mL for optimal sensitivity and 1091·0 pg/mL for specificity using a clinic-ready automated microfluidics-based immunoassay.

**Interpretation:**

These results confirm CXCL10 as a robust nasopharyngeal biomarker of viral respiratory infection and support host response-based screening followed by metagenomic sequencing of CXCL10-high samples as a practical approach to incorporate clinical samples into pathogen discovery and surveillance efforts.

**Funding:**

National Institutes of Health, the Hartwell Foundation, the Gruber Foundation, Fast Grants for COVID-19 research from the Mercatus Center, and the Huffman Family Donor Advised Fund.

## Introduction

The COVID-19 pandemic has renewed the imperative to expand surveillance for unrecognised or emerging pathogens.^1,2^ For respiratory viruses, proposed approaches include isolating viruses from animal sources, identifying unexpected viruses in pooled human respiratory samples, and surveillance for outbreaks, as in the unexplained pneumonia surveillance that led to the initial identification of SARS-CoV-2.^3-5^ These methods can be coupled with metagenomic sequencing for viral identification and molecular epidemiology.^6,7^ However, although screening animal or pooled human samples might identify unrecognised viruses, this approach does not specifically identify viruses capable of causing human disease. Monitoring for unexplained outbreaks targets human pathogens but might find emerging viruses too late—ie, after an epidemic has already begun.

A complementary approach would be to routinely search for missed infections in symptomatic patients. The Yale New Haven health-care system, along with many others, offers multiplex respiratory virus testing on nasopharyngeal swabs, and no virus is identified in most tested samples.^8^ These samples are possibly an important source of unrecognised pathogens. However, the large volume of samples (which can be hundreds per week) and low probability of finding an unexpected pathogen means that performing costly analyses, such as metagenomic sequencing, on every sample is prohibitively inefficient.

A potential solution to this challenge is to leverage the innate immune response to direct pathogen discovery efforts towards samples most likely to contain missed infections. Viral PCR tests can only detect viruses for which the tests were designed, whereas the innate immune system recognises molecular features common to all pathogens of a given class.^9^ For example, sensors in nasal epithelial cells detect features of viral RNA that are distinct from host RNA, but common to many different viruses.^10^ Viral RNA sensing leads to rapid production of interferon and interferon-stimulated genes, a network of molecules designed to block viral replication, many of which are not expressed in a healthy state.^11^ Previous work shows that nasal interferon response can serve as a biosignature of diverse viral respiratory infections, including SARS-CoV-2.^12-15^ We also showed that elevation of nasopharyngeal CXCL10, a chemokine produced during the interferon response, is a sensitive biomarker of viral respiratory infection.^13^

Here, we aimed to determine whether nasopharyngeal CXCL10 screening could be used as a strategy to identify the subset of samples most likely to contain undiagnosed viruses. We also used RNA sequencing (RNA-Seq) and cytokine profiling to compare the host response to infection in samples discovered in the screen and samples that were known to be virus positive or virus negative.

## Methods

### Study design

In this pathogen surveillance and detection study, we analysed residual nasopharyngeal swab samples from patients in the Yale New Haven health-care system who underwent clinical virology testing using a multiplex PCR panel at the Yale New Haven Hospital Clinical Virology Laboratory (New Haven, CT, USA), as previously described.^8^ All samples collected during two time periods were eligible for inclusion; January 23–29, 2017 was selected because this period had high virus circulation and March 3–14, 2020 was selected because this period was the interval between first cases of COVID-19 in our region and availability of SARS-CoV-2 testing at the Yale New Haven Hospital Clinical Virology Laboratory. Nasopharyngeal innate immune responses in screened samples were compared with known virus-positive or virus-negative samples (controls) using transcriptome analysis and cytokine profiling. We also tested paired samples from patients positive for SARS-CoV-2 described in a previous study,^16^ from the peak of infection (samples with highest viral load) and the end of infection (defined as the first sample with SARS-CoV-2 quantitative RT-PCR (RT-qPCR) N1 PCR cycle threshold value >30). Metagenomic sequencing and analysis was performed on discovered viruses to evaluate molecular epidemiology.

For the 2017 time period, RNA-Seq and analysis for viral reads was performed on samples with CXCL10 higher than 1 ng/mL. For the 2020 time period, CXCL10 testing and RT-qPCR for SARS-CoV-2 were performed on all samples.

Demographic and clinical data associated with samples was obtained from the electronic medical record using manual chart review for respiratory virus panel (RVP)-negative samples from 2017 and CXCL10-high RVP-negative samples from 2020, and automated data extraction from the Observational Medical Outcomes Partnership Data Repository was used for RVP-negative samples from 2020. The study protocol was reviewed and approved by the Yale Human Investigation Committee (protocol 2000027656) and was determined to not require specific patient consent.

### Procedures

Viral transport media associated with the nasopharyngeal swab was stored at −80°C at the time of testing, then thawed on ice for CXCL10 measurements and aliquoted and stored at −80°C for use in other analyses, including transcriptomics and cytokine profiling. CXCL10 concentration was measured using an ELISA according to manufacturer’s instructions (R&D Systems, Minneapolis, MN, USA). For the 2017 screen, samples with CXCL10 higher than 150 pg/mL were identified as CXCL10 high for further analysis.

For the 2017 screen, RNA-Seq data were used to map viral and microbial reads using the Chan Zuckerberg ID platform^17^ and for host transcriptomic analysis. Host and microbial RNA-seq was also performed on nasopharyngeal samples from patients with rhinovirus, CoV-NL63, or SARS-CoV-2, or from asymptomatic health-care workers.

Multiplex cytokine measurements were performed using the BioPlex 200 HD71 71-plex Human Cytokine Array–Chemokine Array (Eve Technologies, Calgary, AB, Canada). CXCL10 cutoffs for screening on a clinic-ready platform were defined using an automated microfluidics-based immunoassay (ProteinSimple, R&D Systems, Minneapolis, MN, USA) to measure CXCL10 concentrations in a previously described sample set.^13,18^

Metagenomic sequencing and phylogenetic analysis of SARS-CoV-2 and influenza C virus isolates was performed as previously described.^19,20^ The sample containing reads from influenza C virus was inoculated onto primary human nasal epithelial cells. On day 7 after inoculation, cell morphology was imaged with an Olympus CKX52 inverted microscope and RNA was isolated from cell supernatants for influenza C virus RT-qPCR. Detailed methods are described in appendix (pp 2–4). Genomes used for phylogenetic analysis are provided in Mendeley Data.

### Statistical analysis

GraphPad Prism (version 9.3.1; GraphPad Software, San Diego, CA, USA) was used for correlation analysis of CXCL10 values from different assay platforms. We used SAS Studio (version 3.7) for the χ^2^ test and R (version 3.5.1) for Fisher’s exact test to compare patient groups, and IBM SPSS Statistics (version 28.0.0.0) to generate receiver operating characteristic (ROC) curves.

### Role of the funding source

The funders of the study had no role in study design, data collection, data analysis, data interpretation, or writing of the report.

## Results

**Figure 1:**
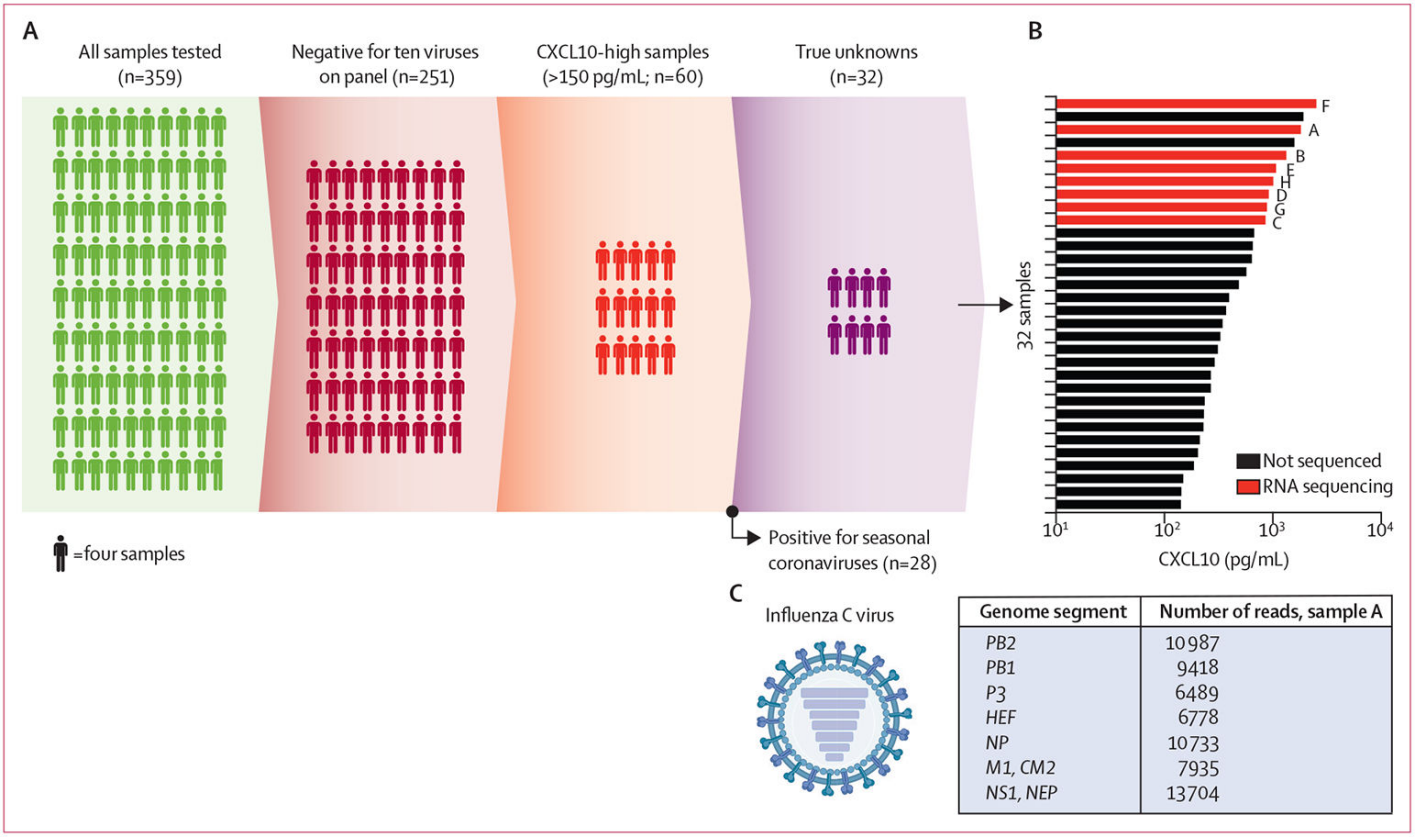
Host response-based screen for undiagnosed infections (week 4, January, 2017) (A) The screening process for samples tested with the respiratory virus panel. True unknowns were negative for the ten viruses on the respiratory virus panel and parainfluenza virus 4 and four seasonal coronaviruses (not included in the respiratory virus panel). (B) Nasopharyngeal CXCL10 concentrations for virus PCR-negative samples, showing those selected for RNA sequencing (samples A–H) based on CXCL10 (>1 ng/mL) and sufficient RNA content. (C) Reads in sample A map to each of the seven gene segments of the influenza C virus genome.

Overall, 359 nasopharyngeal samples were tested for viruses during January 23–29, 2017, and 651 samples were tested during March 3–14, 2020. In 2017, 251 (70%) of 359 samples tested negative for ten viruses (rhinovirus, influenza A and B, parainfluenza 1–3, respiratory syncytial virus A and B, human metapneumovirus, and adenovirus) on the multiplex PCR respiratory virus panel (RVP; figure 1; appendix p 9). RVP-negative samples were from children and adults in inpatient and outpatient settings, with 134 (53%) presenting with respiratory symptoms and 84 (34%) with a history of chronic respiratory illness. The characteristics of all patients who provided samples are shown in the appendix (p 10). 60 (24%) of 251 RVP-negative samples had CXCL10 higher than 150 pg/mL, which corresponded to a sensitivity and specificity of approximately 80% for respiratory virus detection in our previous study.^13^ Additional testing of 2017 samples for common respiratory viral pathogens (coronaviruses [CoV-OC43, CoV-229E, CoV-NL63, and CoV-HKU1] and parainfluenza 4) that were not included on the RVP in 2017, revealed that 28 (47%) of 60 CXCL10-high samples were positive for seasonal coronaviruses (figure 1A). Thus, 223 (89%) of 251 samples were PCR-negative for 15 viruses and, of these, CXCL10-based screening identified 32 (13%) samples for further analysis.

CXCL10-high samples were more likely to be from young patients (eg, three [9%] of 32 CXCL10-high samples *vs* four [2%] of 191 CXCL10-low samples were from patients younger than 5 years) and those who had fatigue listed as a presenting symptom (five [16%] *vs* three [2%]; appendix p 11). Otherwise, clinical and demographic features were not significantly different between CXCL10-high samples and CXCL10-low samples, indicating that nasopharyngeal CXCL10 screening identifies RVP-negative samples not easily identified by clinical features.

Of the 32 CXCL10-high samples with no virus detected (true unknowns), eight (25%) of the samples with highest CXCL10 concentrations (>1 ng/mL) and sufficient RNA were selected for RNA-Seq, since CXCL10 concentration correlated directly with likelihood of respiratory virus detection by PCR in our previous study (figure 1B).^13^ Read mapping to viral reference sequences in GenBank revealed that one sample had more than 60 000 reads mapping to the influenza C virus across all seven genome segments (figure 1C). This sample was taken from a child younger than 5 years with acute respiratory illness (sample A; table). Influenza C virus was also detected in this sample using RT-qPCR but was not detected in the other 31 CXCL10-high samples. Primary human nasal epithelial cells inoculated with sample A showed cell–cell fusion on day 7 after inoculation; at this time influenza C virus RNA was also detectable by PCR in the cell culture supernatant (appendix p 5). Together, these results support the presence of influenza C virus in the original sample and show cytopathic effects on primary human nasal epithelial cells. Phylogenetic analysis placed this isolate within the São Paolo lineage (appendix p 5). Comparison with other influenza C virus sequences in the Global Initiative on Sharing Avian Influenza Data showed high similarity to influenza C virus circulating in Hong Kong and Japan from 2014 to 2018 (appendix p 5). Genomes used for phylogenetic analysis are provided in Mendeley Data.

Although no other viruses were identified by RNA-Seq, review of medical records revealed that two samples were from young adults (aged 20–25 years) diagnosed with acute Epstein-Barr virus infection (sample G) and acute cytomegalovirus infection (sample H) on the basis of serology, blood smear, and plasma PCR (for Epstein-Barr virus) at the time of swab collection (table). Thus, acute viral infections were identified in three (samples C, G, and H) of the eight CXCL10-high samples.

Analysis of microbial RNA using the Chan Zuckerberg ID platform^17^ confirmed the presence of influenza C virus in sample A and revealed RNA reads from bacterial pathobionts *Haemophilus influenzae* or *Moraxella catarrhalis* in four (50%; samples A–C, and F) of eight samples, with abundant bacterial RNA in two of these samples (A and B; >10^5^ reads per million [rpm]; table). Since these bacteria can cause illness on their own or as copathogens with viruses, these microbes might have caused or contributed to patient symptoms. No pathogens were identified in samples D and E, which were from patients in the intensive care unit with complex clinical courses. No other pathobionts were detected (>10^4^ rpm) in the sequenced samples.

**Figure 2:**
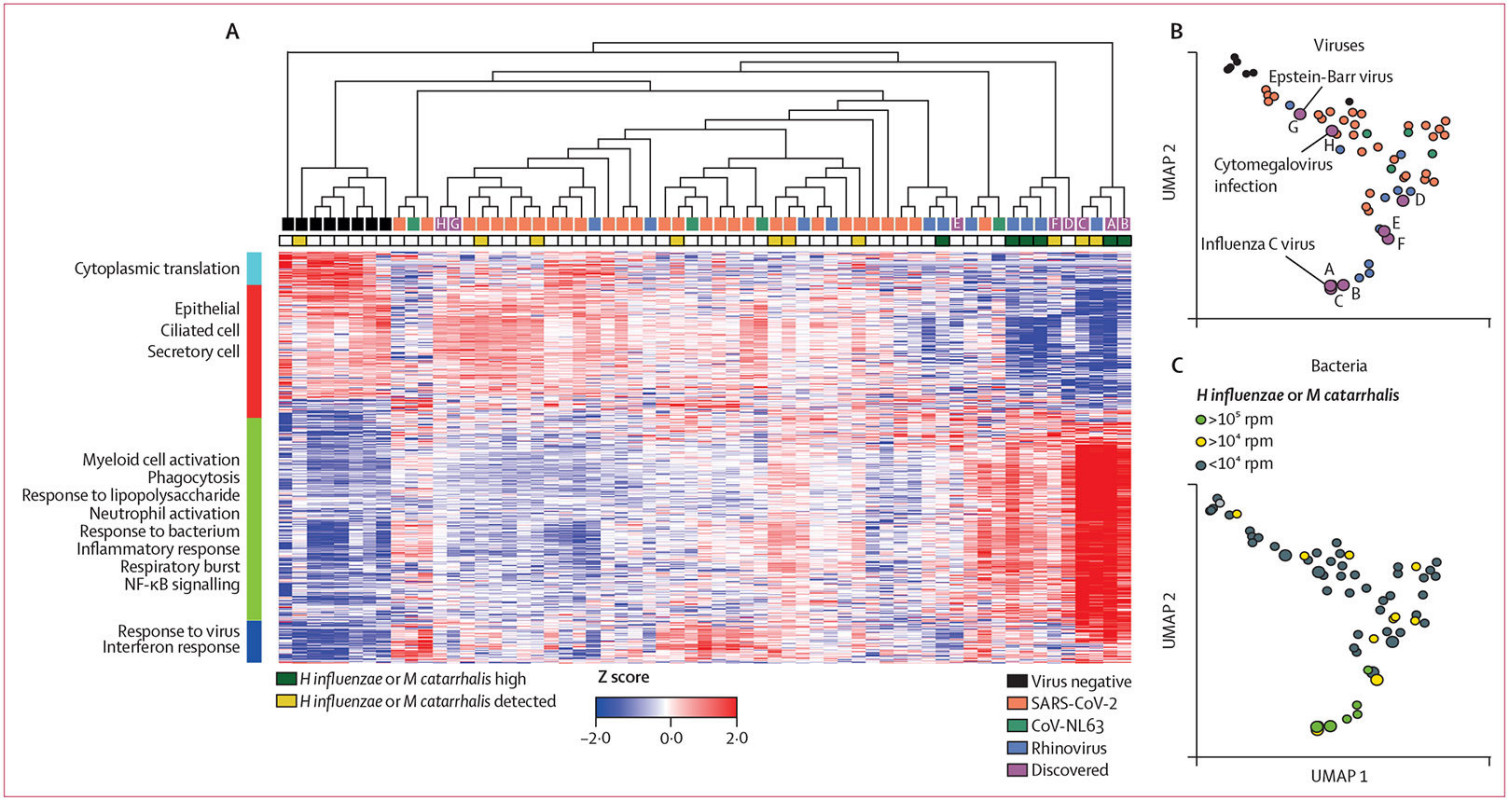
Unsupervised clustering of 53 known virus-positive and virus-negative control samples and discovered samples from the 2017 screen based on transcriptomic signatures (A) Heatmap showing unsupervised clustering of samples based on top differentially expressed genes across sample groups (p≤0·005; n=2678 genes; Qlucore Omics Explorer; version 3.7). Gene Ontology biological functions for major gene clusters are indicated on the left. (B) UMAP plot showing the relationship between transcriptomes of 53 known virus-positive and virus-negative samples and those discovered in the 2017 screen. Virus-positive samples are orange (SARS-CoV-2), green (CoV-NL63), and blue (rhinovirus); discovered samples are purple; and virus-negative samples are black. (C) UMAP plot indicating samples with moderate or high bacterial pathobiont (*Haemophilus influenzae* or *Moraxella catarrhalis*) concentrations. rpm=reads per million. UMAP=Uniform Manifold Approximation and Projection.

To gain further insight into the types of infections and nasopharyngeal host responses associated with CXCL10 elevation, we compared microbial reads and host transcriptional responses in the eight discovered samples with virus-negative samples and samples positive for rhinovirus, seasonal coronavirus CoV-NL63, or SARS-CoV-2 (figure 2, appendix pp 12–14). Unsupervised clustering based on differential gene expression segregated nasopharyngeal samples into three immunological patterns: virus-negative controls enriched for airway epithelial genes without induction innate immune responses (left of figure 2A); virus-positive samples enriched for airway epithelial genes and an interferon response signature (centre of figure 2A); and samples with heightened innate immunity with enrichment of leukocyte transcripts and genes associated with neutrophil activation, such as NF-κB signalling, phagocytosis, and respiratory burst (right of figure 2A).

Consistent with heatmap data, Uniform Manifold Approximation and Projection (UMAP)^21^ based on host differentially expressed transcripts also showed segregation of no infection, only viral infection, and pathobiont-high samples (figure 2B, C). In heatmap and UMAP data, the CXCL10-high RVP-negative samples clustered with the known virus-positive samples rather than virus-negative samples. The host signature from patients with acute Epstein-Barr virus (sample G) or acute cytomegalovirus (sample H) were closer to negative controls than other virus-positive samples, showing an epithelial cell signature and a moderate interferon response signature. Three samples (D, E, and F) from patients with severe respiratory illness and no pathogen identified showed an interferon and inflammatory response signature (figure 2; table). The three RVP-negative samples from outpatients with high pathobiont amounts (A, B, and C), including the sample positive for influenza C virus, showed pronounced heightened innate immunity and neutrophil infiltration signatures and clustered with known virus-positive samples that contained high pathobiont amounts (figure 2A; table). Gene Ontology pathways associated with each gene cluster are listed in figure 2A and top 20 Gene Ontology Biological Processes are listed in the appendix (pp 15–16). A complete list of genes in each cluster is provided in Mendeley Data.

These data link nasopharyngeal interferon response signatures with the presence of viral respiratory infections and provide additional granularity by revealing at least two distinct host response patterns in CXCL10-high samples, a largely epithelial pattern and a neutrophil enriched pattern (figure 2A). Notably, most known virus-positive samples and discovered samples with the neutrophil enriched pattern had an intermediate (10^4^–10^5^ rpm) or high (>10^5^ rpm) number of reads from bacterial pathobionts *H influenzae* or *M catarrhalis* (figure 2A; appendix pp 12–14) suggesting that this distinct neutrophil-enriched nasopharyngeal inflammation pattern might signal high amounts of bacterial pathobionts.

**Figure 3:**
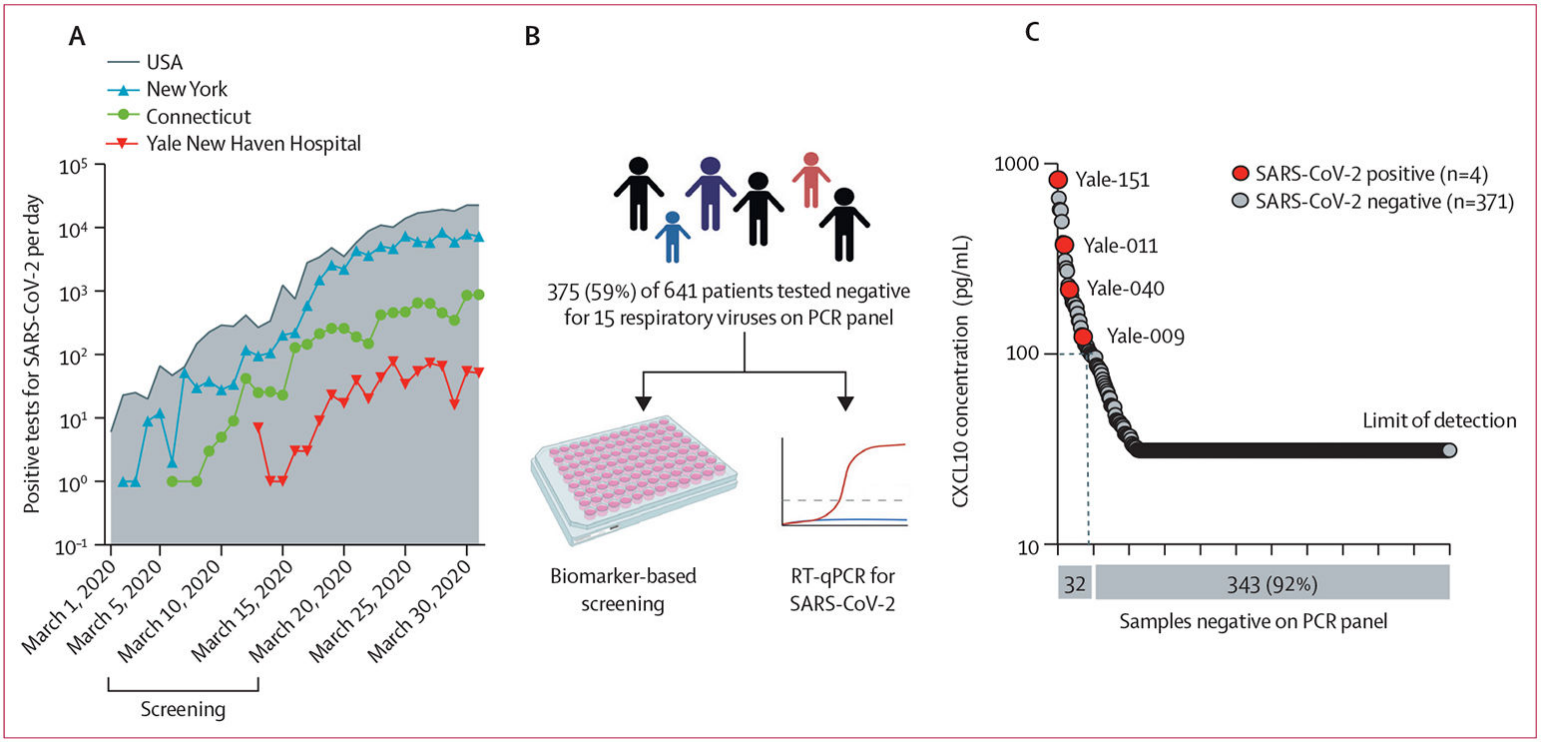
Discovery of four undiagnosed cases of SARS-CoV-2 in Connecticut, USA (March, 2020) (A) SARS-CoV-2 positive tests during March, 2020, according to publicly available data.^22-24^ SARS-CoV-2 testing began at Yale New Haven Hospital on March 13, 2020. Screening was performed between March 3 and March 14, 2020. (B) In all negative samples on the multiplex viral PCR panel, CXCL10 was measured by ELISA (biomarker-based screening) and SARS-CoV-2 (N1 gene) was measured by RT-qPCR. (C) Four discovered SARS-CoV-2 positive samples shown (red) with corresponding NextStrain codes. The dotted line indicates the proportion of samples with nasopharyngeal CXCL10 concentration of 100 pg/mL or higher.

To further evaluate the usefulness of CXCL10 in enriching for nasopharyngeal samples containing undiagnosed respiratory viruses, we evaluated samples at the start of the COVID-19 pandemic. The Yale New Haven health-care system includes patients from southern Connecticut and eastern New York, USA, where the first reported case occurred on March 2, 2020 (figure 3A).^22-24^ SARS-CoV-2 testing began at the Yale New Haven Hospital on March 13, 2020. Between March 3 and March 14, 375 (59%) of 641 nasopharyngeal swab samples tested negative for 15 viruses on the RVP, which did not include SARS-CoV-2. 32 (9%) of 375 samples had CXCL10 concentrations ranging from 100 pg/mL to 1000 pg/mL. When we tested these samples for SARS-CoV-2 by RT-qPCR, we found that four of these were positive for SARS-CoV-2; whereas, 343 (92%) samples had CXCL10 concentrations lower than 100 pg/mL and all were SARS-CoV-2 negative (figure 3C). Data extraction from the electronic medical record showed no significant differences in the demographic or clinical characteristics between four patients testing positive and 371 patients testing negative for SARS-CoV-2; however, CXCL10 elevation was statistically significant, and a distinguishing feature was found in all four (100%) SARS-CoV-2-positive samples and 28 (8%) of 371 SARS-CoV-2-negative samples (p=4·4×10^−5^; appendix p 17).

Metagenomic sequence analysis revealed that the four discovered SARS-CoV-2 isolates were epidemiologically relevant, since all were genetically distinct and belonged to different lineages and sublineages, indicating independent introductions to the region of the Yale New Haven Hospital (appendix p 6). These data are consistent with previous studies showing that SARS-CoV-2 entered the regions of Connecticut served by our health-care system via multiple independent lines of transmission before March 14, 2020.^19^

Chart review for the 28 RVP-negative SARS-CoV-2-negative samples with elevated CXCL10 from March, 2020, revealed an infectious cause of disease in one patient—a child younger than 5 years diagnosed with acute cytomegalovirus and acute Epstein-Barr virus at the time of the nasopharyngeal swab collection, based on clinical presentation, serology, and elevated lymphocyte count in the peripheral blood. Of 375 RVP-negative patients screened between March 3 and March 14, 16 (4%) underwent serological testing for Epstein-Barr virus or cytomegalovirus, or both, but no other patients were diagnosed with these infections.

**Figure 4:**
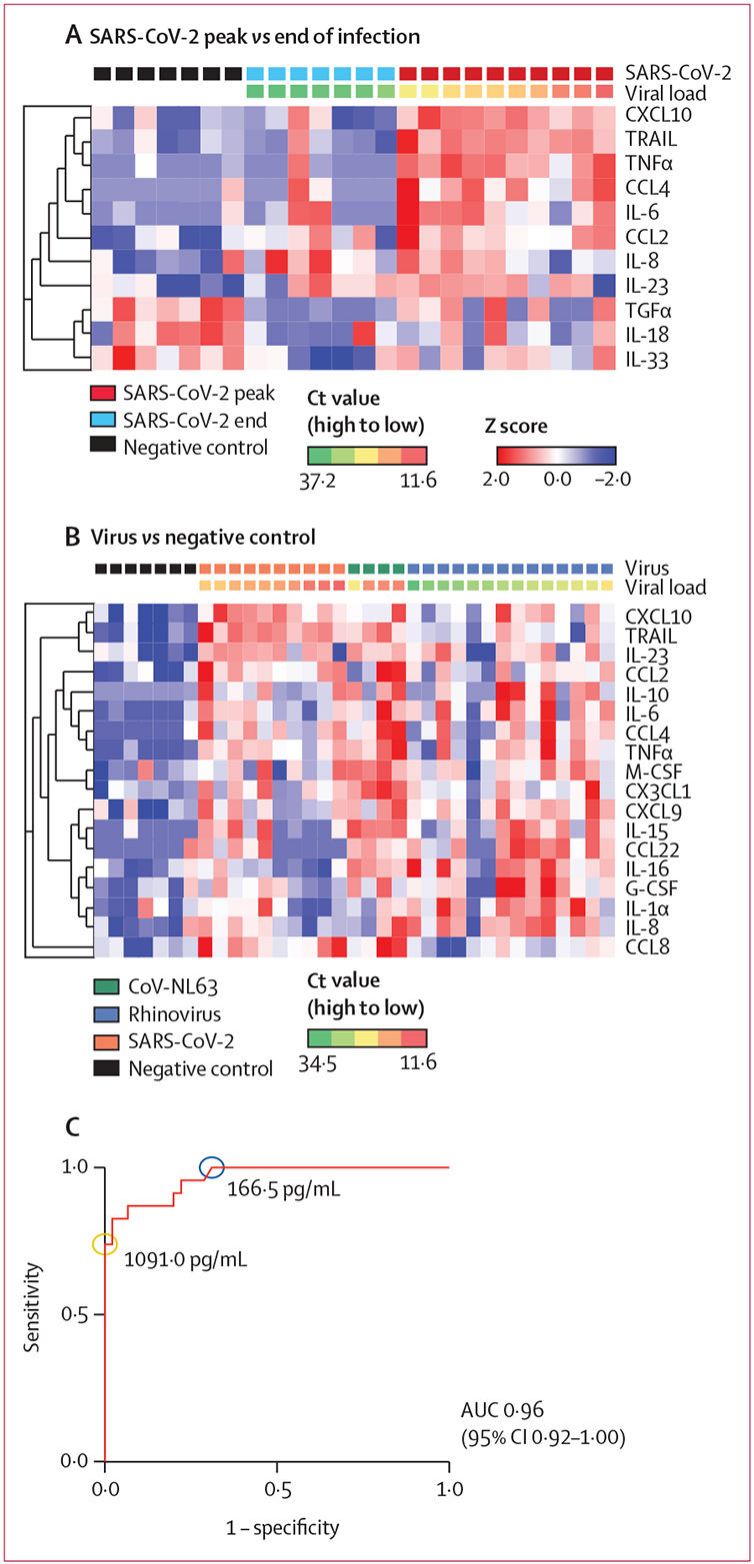
Nasopharyngeal cytokine expression in virus-positive and virus-negative samples Proteomic signature of top differentially expressed cytokines in patients with SARS-CoV-2 at the peak viral load versus end of infection and virus-negative health-care workers, using a multigroup comparison (A), and virus-positive patients and virus-negative health-care workers based on a two-group comparison (B). Cytokines were measured using a 71-plex immunoassay (Eve Technologies, Alberta, Canada). Heatmaps show cytokines clustered by coexpression and arranged by viral load (low Ct represents high viral load and high Ct represents low viral load). Z score represents SD from the mean. Peak of infection is defined as the longitudinal sample with highest viral load, and the end of infection is defined as the first sample with a Ct value higher than 30. (C) Receiver operating characteristic for CXCL10 concentrations based on values from an automated microfluidic assay, using a sample set in which the prevalence of viral respiratory infection is 34%. Cutoffs predicted to maximise sensitivity (blue) or specificity (yellow) for respiratory virus detection are shown. AUC=area under the curve.

We wanted to understand the wide range of nasopharyngeal CXCL10 concentrations in SARS-CoV-2-positive samples and explore additional nasopharyngeal biomarkers of viral infection that might substitute or improve CXCL10-based screening, since previously we showed a direct correlation between viral load and nasopharyngeal CXCL10 concentration for SARS-CoV-2 and rhinovirus.^13,16^ To further explore the relationship between nasopharyngeal cytokines and viral load, we performed a multiplex cytokine immunoassay using paired longitudinal samples from the peak versus end of SARS-CoV-2 infection described in a previous study.^16^ CXCL10 and other interferon-induced cytokines (such as TRAIL) and proinflammatory cytokines (such as TNFα and IL-6) were elevated when the viral load was at its peak (lowest Ct value), whereas towards the end of infection, cytokine patterns more closely matched those seen in virus-negative controls (figure 4A, appendix p 19). Notably, IL-33, TGFα, and IL-18 were depleted in end of infection samples compared with the negative controls, indicating a difference in cytokine profile between resolving infection and baseline innate immune status.

Next, we evaluated cytokine patterns and relationship with viral load for diverse respiratory viral infections (figure 4B, appendix p 19). Consistent with transcriptomic data, compared with negative controls, diverse virus-positive samples showed enrichment of cytokines associated with the interferon response and antiviral immunity. The two interferon-induced cytokines most significantly associated with viral infection were CXCL10 (p=2×10^−5^) and TRAIL (p=1·71×10^−5^; figure 4B; appendix p 7). For each virus, CXCL10 and TRAIL concentrations showed a positive correlation with viral load (figure 4A, B). Unsupervised clustering of these samples with discovered samples from the 2017 screen showed similarities with transcriptome-based clustering (figure 2; appendix p 8).

To facilitate translation to clinical use, we measured CXCL10 using an automated microfluidics-based immunoassay.^18^ We used a previously described sample set with known respiratory virus positivity of 34%.^13^ Although the absolute CXCL10 values were slightly different, measurements from the microfluidic assay correlated with data from the bead-based immunoassay used in our previous study (r 0·9422, 95% CI 0·9051-9650) and with ELISA assays used for screening in this study (r 0·9231, 0·8422–0·9631; appendix p 8). Using the automated assay, the ROC curve for prediction of viral infection from nasopharyngeal CXCL10 concentration had an area under the curve of 0·964 (95% CI 0·92–1·00; figure 4C). The cutoffs to identify virus-positive samples using this platform were 166·5 pg/mL for optimal sensitivity and 1091·0 pg/mL for specificity.

## Discussion

This study presents an efficient pathogen surveillance strategy using respiratory swabs from symptomatic patients that have tested negative on a standard diagnostic RVP. The challenge of using negative samples for surveillance is that the sample pool is large, but the yield is low. Cough, fatigue, and other symptoms that lead to respiratory virus testing have numerous possible non-infectious disease causes, thus, searching for undiagnosed pathogens in every patient becomes inefficient and cost prohibitive. Here, we show that screening for a single cytokine in nasopharyngeal samples identifies a small proportion of total samples that are most likely to contain undiagnosed infections.

Improved efficiency is clear from SARS-CoV-2 PCR and CXCL10 testing of samples from the start of the COVID-19 pandemic. Of 375 samples negative for other respiratory viruses, we found four SARS-CoV-2-positive samples, all with elevated CXCL10. These samples were not distinguishable from SARS-CoV-2-negative samples by other clinical or demographic characteristics. If we used CXCL10 testing as a prescreen to designate which samples to test with viral PCR, we could have identified all undiagnosed SARS-CoV-2 infections by performing viral PCR on 32 samples rather than 375, thus reducing the number of samples undergoing PCR testing and associated costs by more than 90%. For detecting unexpected pathogens using metagenomic sequencing, this increase in efficiency is more impactful, since metagenomic sequencing is more complex, time consuming, and costly than PCR.

Host response-based screening is an attractive strategy for surveillance of emerging viruses. Since this approach relies on immune recognition of features common to many viruses, it requires no previous knowledge of the pathogen. Host response-based approaches could also be used to identify zoonotic pathogens, as shown in a 2020 study,^25^ in which a novel picornavirus was discovered in zebrafish (*Danio rerio*) after investigators observed an interferon response. Since nasopharyngeal samples represent the site of active viral replication at the start of infection, they offer a unique advantage over other sample types (eg, blood) because pathogens can be identified, sequenced, and cultured directly from the same sample used for screening.

Our data support the use of nasopharyngeal CXCL10 as a biomarker in host response-based screening for respiratory viruses and provide a method with relevant cutoffs to incorporate automated CXCL10-based screening for unexpected infections into the clinical workflow. Notably, the innate immune response is dynamic and might become less robust as the viral load declines, which needs to be considered when using nasopharyngeal biomarkers. Thus, host response-based screening might not capture every viral infection, particularly when the viral load is low. However, the correlation between viral load and nasopharyngeal cytokine concentration offers advantages for virus discovery, because samples identified by robust cytokine responses are likely to have high enough viral loads to enable further analyses.

Although the focus of this study was detecting undiagnosed respiratory viruses, we also found three instances of elevated nasopharyngeal CXCL10 in patients with acute cytomegalovirus or acute Epstein-Barr virus, suggesting that nasopharyngeal CXCL10 might be a biomarker for these infections, which acutely infect the respiratory tract, among other sites, but are usually diagnosed by serology.^26,27^ Furthermore, we found a distinct nasopharyngeal transcriptomic signature in some CXCL10-high samples associated with neutrophil infiltration and high amounts of airway bacterial pathobionts. These findings suggest the potential for defining useful nasopharyngeal biomarker signatures that show additional infection types.

Finally, to translate this approach to clinical use, we defined cutoffs for nasopharyngeal CXCL10-based screening using an automated assay that is already in clinical use for laboratory-developed tests. We used a previously described sample set from December, 2017,^13^ in which 34% of the samples are respiratory virus PCR positive. Based on testing practices in 2017, patients were tested only at initial presentation in this sample set, presumably close to the start of illness when viral load was the highest. Thus, these cutoffs would be most relevant to samples collected under similar circumstances. Instituting screening would require a system for curating samples that test negative on the RVP rather than discarding samples, after which CXCL10-based screening could be performed in real time or retrospectively on frozen stored samples to maximise convenience and efficiency, followed by metagenomic sequencing of CXCL10-high samples. Thus, we describe a straightforward workflow that can be readily implemented in clinical and public health laboratories.

In conclusion, we show a practical and efficient strategy for screening patient samples to identify those most likely to contain missed viral infections. This approach opens up new avenues in the global effort to scale up surveillance for pathogens that represent unrecognised threats to human health.

## Supplementary Material

1

## Figures and Tables

**Table:** Clinical presentation and pathobiont reads of patients with CXCL10-high nasopharyngeal samples discovered in January, 2017

	Age, years	Sex	Clinical history	Pathobiont rpm (genome coverage, %)
A	0–5	Male	Acute respiratory illness and cough*	*Haemophilus influenzae* 5·6 rpm × 10^5^ (99·9%), *Moraxella catarrhalis* 0·3 rpm × 10^5^ (13·1%)
B	0–5	Female	Acute respiratory illness and fever*	*H influenzae* 0·3 rpm × 10^5^ (1·4%), *M catarrhalis* 3·4 rpm × 10^5^ (64·3%)
C	55–60	Male	Acute respiratory illness, cough, and fever*	*H influenzae* 0·3 rpm × 10^5^ (4·8%)
D	45–50	Male	Acute respiratory failure; intensive care unit	..
E	60–65	Female	Chronic obstructive pulmonary disease, lung cancer, and fever; intensive care unit	..
F	70–75	Female	Chronic obstructive pulmonary disease exacerbation, supplemental oxygen	*H influenzae* 0·3 rpm × 10^5^ (24·6%)
G	20–25	Male	Fever and dyspnoea; diagnosis of acute Epstein-Barr virus infection†	..
H	20–25	Female	Fever and rash; diagnosis of acute cytomegalovirus infection‡	..

rpm=reads per million.

* Outpatient (sample discovered to contain influenza C virus).

† Serology consistent with acute Epstein-Barr virus, plasma PCR-positive for Epstein-Barr virus, and complete blood count showed 47% atypical lymphocytes.

‡ Serology consistent with acute cytomegalovirus infection and complete blood count showed 75% lymphocytes with atypical lymphocytes.

## Data Availability

RNA sequencing data from patient nasopharyngeal samples have been deposited to the Database of Genotypes and Phenotypes (accession codes phs002433.v1.p1 and phs002442.v1.p1). The influenza C virus sequencing data were deposited to GenBank (OK625706-OK625712) and the SARS-CoV-2 sequencing data were deposited to the Global Initiative on Sharing Avian Influenza Data (EPI_ISL_416423, EPI_ISL_435720, EPI_ISL_428250, and EPI_ISL_428250). Extended data files are shared in Mendeley Data (http://dx.doi.org/10.17632/6g9n6xdrzr.1).

## Research in context

### Evidence before this study

We searched PubMed from database inception to Dec 17, 2021, for previous studies on nasal immune biomarkers of viral respiratory infection using the terms “nasal or naso* or nose” AND “host response or gene expression” AND “respiratory virus” AND “diagnostic”, with no language restrictions. Of 625 search results, 24 studies showed nasal interferon responses during respiratory virus infections, with four showing diagnostic use of nasal interferon signatures for viral infection. This search included a previous study from our group showing that interferon-induced cytokine CXCL10 is a sensitive nasopharyngeal biomarker of viral respiratory infections with known causes. However, to our knowledge, no previous studies have used nasopharyngeal cytokines to screen for and identify undiagnosed viral respiratory infections.

### Added value of this study

We showed that screening nasopharyngeal swabs for a cytokine produced during the airway antiviral response could enrich for samples containing undiagnosed respiratory pathogens and capture epidemiologically substantial emerging infections, including four genetically distinct isolates of SARS-CoV-2 from the start of the COVID-19 pandemic in March, 2020, in Connecticut (northeastern USA) and an unexpected influenza C infection. We evaluated the relationship between nasopharyngeal CXCL10 concentration and host response patterns measured by transcriptomics and proteomics, and the relationship of nasopharyngeal cytokine concentrations to infection status and viral load. We defined clinically relevant CXCL10 cutoffs to maximise sensitivity or specificity of screening for undiagnosed viruses using a microfluidics-based platform.

### Implications of all the available evidence

This study shows that the nasopharyngeal innate immune response can be leveraged to identify high-yield samples among a large number of respiratory samples negative for common viruses and can be practically applied to incorporate such samples into pathogen discovery and surveillance efforts.
