# Supplementary material for: Nasal host response-based screening for undiagnosed respiratory viruses: a pathogen surveillance and detection study
Source: Lancet Microbe. Author manuscript; Available in PMC 2023 Jan 12. (PMC9835789; doi:10.1016/S2666-5247(22)00296-8)
Supplement: 1 [file NIHMS1861378-supplement-1.pdf]

THE LANCET
Microbe

Supplementary appendix

This appendix formed part of the original submission and has been peer reviewed. We post it as supplied by the authors.

Supplement to: Cheemarla NR, Hanron A, Fauver JR, et al. Nasal host response-based screening for undiagnosed respiratory viruses: a pathogen surveillance and detection study. *Lancet Microbe* 2023; **4:** e38–46.

Supplementary Materials

Supplementary Methods

Supplementary Figures

Figure S1 (related to Fig 1). Primary human nasal epithelial cells 7 days post-inoculation with sample A containing influenza C virus and molecular epidemiology of influenza C isolate.

Figure S2 (related to Fig 3). Phylogenetic analysis of four SARS-CoV-2 isolated identified in March 2020 screen.

Figure S3 (related to Fig 4). P- and Q- (FDR) values for cytokines differentially elevated in virus positive samples compared to virus negative subjects.

Figure S4 (related to Fig 4). Unsupervised clustering of known virus-positive and virus-negative samples and discovered samples from 2017 screen based on cytokine expression patterns.

Figure S5 (related to Fig 4). Correlation of CXCL10 values on different assay platforms.

Supplementary Tables

Table S1 (related to Fig 1 and 3). Respiratory viruses detected by the Yale-New Haven Hospital PCR panel, 2020 and 2017

Table S2 (related to Fig 1). Patient demographics and clinical presentation associated with the 251-nasopharyngeal samples testing negative for respiratory viruses, week 4, January 2017

Table S3 (related to Fig 1 and Table 1). Description of PCR negative samples from week 4, January 2017: CXCL10 negative vs CXCL10 positive

Table S4 (related to Fig 2). Microbial reads, viral PCR Ct values, and clinical features of known virus- negative and virus-positive samples used for transcriptomics analysis.

Table S5 (related to Fig 2). Top Gene Ontology Biological Processes associated with clusters highlighted in Fig 2.

Table S6 (related to Fig 3). Comparison of patient features and CXCL10 levels for samples testing SARS-CoV-2-negative or positive samples in the March 2020 screen.

Table S7 (related to Fig 3). Summary statistics for MinION sequencing of SARS-CoV-2 positive samples.

Table S8 (related to Fig 4) Clinical and microbiological data for samples used for proteomic analyses.

Supplementary Methods

Human experimental guidelines approval statement

Residual nasopharyngeal samples from clinical testing were obtained from the Yale-New Haven Hospital Clinical Virology Laboratory and medical records were reviewed, followed by de-identification. Prior to data de-identification, discovered positive SARS-CoV-2 cases were reported to health care providers according to IRB-approved protocol #2000027656 with oversight from the Yale Human Investigations Committee.

Clinical samples

Nasopharyngeal swabs were frozen at the time of clinical testing and stored at -80°C until use. To perform CXCL10 screening, samples were thawed on ice, aliquoted, and used for ELISA, cytokine profiling, and/or RNASeq analysis as follows. Clinical information including age, sex, virology and microbiology results, and specific features of clinical course including presenting symptoms, hospital admission and length of stay, was extracted from the electronic medical record and recorded, after which samples were assigned a study code and de-identified. For determining the CXCL10 cutoffs to optimize sensitivity and specificity, a previously- described sample set of 68 samples was used, of which 23 were respiratory virus-positive (prevalence 34%)[1].

Clinical virology testing

For testing by the YNHH Clinical Virology Laboratory, NP swabs were placed in viral transport media (BD Universal Viral Transport Medium) immediately upon collection. Samples (200 µL) were subjected to total nucleic acid extraction using the NUCLISENS easyMAG platform (BioMérieux, France). The 10-virus PCR panel was performed as described previously[1]. CXCL10- high samples from January 2017 were tested for four coronaviruses and PIV4 by PCR as described previously[1]. The 15-virus PCR panel use in March 2020 included updated rhinovirus PCR detection and inclusion of 4 seasonal coronaviruses and parainfluenza virus 4[1-3]. YNHH testing for SARS-CoV-2 was done using N1, N2, and RNAse P primer probe sets with an emergency use authorized assay developed by the CDC[4].

CXCL10 measurements

CXCL10 measurements were performed on each sample in duplicate by ELISA (Cat No: DY266, R&D systems, Minneapolis, MN, USA) and concentrations were calculated from a standard curve on each plate according to manufacturer instructions using GraphPad Prism software. Microfluidics-based immunoassay for CXCL10 was performed using the SimplePlex ELLA microfluidics platform and analyzed by the SimplePlex Explorer software according to the manufacturer's instructions (Protein simple, San Jose, CA, USA)[5]. Results show mean of each sample run in duplicate (ELISA) or triplicate (ELLA).

RNA isolation, Library preparation and RNA Sequencing

We performed ribodepletion RNAseq without low input amplification, which we sought to avoid since low input methods led to amplification of environmental microbes in preliminary studies. RNA was isolated from 140µl of transport medium using the Qiagen Viral RNA isolation kit per manufacturer's instructions (Ref: 52904, Qiagen, Germantown, MD, USA). RNA was quantified using the Agilent 2100 Bioanalyzer Pico RNA Assay. Library preparation was performed using Kapa Biosystem's KAPA HyperPrep Kit with RiboErase (HMR) in which samples were normalized with a total RNA input of 25ng. Libraries were amplified using 15 PCR cycles, validated using Agilent TapeStation 4200 D1000 assay, and quantified using the KAPA Library Quantification Kit for Illumina® Platforms kit. Libraries were diluted to 1.3nM and pooled at 1.25% each of an Illumina NovaSeq 6000 S4 flowcell using the XP workflow to generate 25M read pairs/sample at the Yale Center for Genomic Analysis.

RNASeq data analysis

Low quality reads were trimmed and adaptor contamination was removed using Trim Galore (v0.5.0, https://www.bioinformatics.babraham.ac.uk/projects/trim_galore/). Trimmed reads were mapped to the human reference genome (hg38) using HISAT2 (v2.1.0)[6]. Gene expression levels were quantified using StringTie (v1.3.3b) with gene models (v27) from the GENCODE project[7]. Differentially expressed genes (adjusted p value < 0.05, fold change cutoff = 2) were identified using DESeq2 (v 1.22.1) [8]. Master DEG list used for transcriptomic analyses was compiled by merging in DEGs determined by DeSeq, based on pairwise comparisons of virus-positive groups (RV, CoV-NL63, SARS-CoV-2, RV, pathobiont low) to virus negative controls and pairwise comparisons of each virus positive group (RV vs. SARS-CoV-2. RV vs CoV-NL63, CoV-NL63 vs. SARS-CoV2) (n=5773 DEG). Pathway analysis and upstream regulators of DEG in pairwise comparisons was visualized using Ingenuity Pathway analysis (version 01-16).

Mapping to viral reference genomes

To identify the viral sequences in 2017 RNASeq data, we constructed a hybrid genome consisting of human reference genome (hg38), a curated collection of 16S rRNA sequences from bacteria and archaea in NCBI RefSeq database as of March 30, 2020 (downloaded from https://www.ncbi.nlm.nih.gov/refseq/targetedloci/16S_process/), and a curated collection of viral genomic sequences in NCBI RefSeq database as of March 30, 2020 (downloaded from ftp://ftp.ncbi.nlm.nih.gov/refseq/release/viral).

Then we indexed this hybrid genome for HISAT2 and aligned the RNA-seq reads, which were processed using Trim Galore, to the hybrid genome using HISAT2 (v2.1.0) [9]. To obtain the reliable numbers of reads that were mapped to viral sequences, we only considered high-quality reads with MAPQ >= 60 and excluded reads with 15 or more consecutive polyN bases.

RT-qPCR for influenza C
RNA was isolated from 140µl of cell culture supernatant (in vitro infection) or viral transport medium (clinical samples) as described above, followed by cDNA synthesis using iScript cDNA synthesis kit (BioRad, Hercules, CA, USA). qPCR was performed using SYBR green iTaq universal (BioRad) per manufacturer's instructions, using the following PCR primers:
ICV S7 gene (F- TCCAAAATGTCCGACAAAACAGT, R- TGCATTTCAGTGCATGTGTCT)
ICV M2 gene (F- GTCTCAGAAAGTGGAAGAACAGC, R- CCAAGGCCAGTAATACCAGCA)

In vitro infections.
Primary human nasal epithelial cells (Promocell, Germany) were grown in conventional culture using BEGM media (Lonza, Walkersville, MD, USA), then inoculated with sample A or viral transport medium only. After 7 days of incubation, micrographs were taken to record cell appearance and supernatant was used for RNA isolation and influenza C RT-qPCR.

SARS-CoV-2 screening by PCR
For screening of 641 respiratory virus panel negative samples from 2020 for SARS-CoV-2 RNA, eluates from easyMag RNA extraction were screened using the US CDC 2019-nCoV N1 primer probe set or the E gene Sarbeco primer probe set, using the following reaction conditions as described previously(IDT, Coralville, Iowa)[10]. We used the Luna Universal Probe One-step RT-qPCR kit (New England Biolabs, Ipswich, MA, USA) with 5 µL of RNA and primer and probe concentrations of 500 nM of forward and reverse primer, and 250 nM of probe. PCR cycler conditions were reverse transcription for 10 minutes at 55°C, initial denaturation for 1 min at 95°C, followed by 40 cycles of 10 seconds at 95°C and 20 seconds at 55°C on the Biorad CFX96 qPCR machine (Biorad, Hercules, CA, USA). PCR-positive samples were confirmed by the YNHH clinical laboratories using an EUA clinical assay using the CDC-developed primer sequences.

SARS-CoV-2 sequencing and analysis
SARS-CoV-2 positive samples were processed for next-generation sequencing as previously described[11]. Total nucleic acid was subjected to cDNA synthesis using SuperScript IV VILO Master Mix (ThermoFischer Scientific, MA, USA) according to the manufacturer's protocol. cDNA was used as input into a highly multiplexed amplicon generation approach for sequencing on the Oxford Nanopore Technologies MinION (ONT, Oxford, UK)[12]. Samples were barcoded using the Native Barcoding Expansion Pack (ONT, Oxford, UK), multiplexed, and sequenced using R9.4.1 flow cells (ONT, Oxford, UK). The RAMPART software from the ARTIC Network was used to monitor each sequencing run. Runs were stopped when sufficient depth of coverage was achieved to accurately generate a consensus sequence. Following the completion of each sequencing run, raw reads (.fast5 files) were basecalled using Guppy high-accuracy model (v3.5.1, ONT, Oxford, UK). Basecalled FASTQ files were used as input into the ARTIC Networks consensus sequence generation bioinformatic pipeline (https://artic.network/ncov-2019/ncov2019-bioinformatics-sop.html). Variants to the reference genome were called with nanopolish[13]. Stretches of the genome that were not covered by 20 or more reads were represented by stretches of NNN's.

Phylogenetic analysis
To infer the evolutionary history and origins of the SARS-CoV-2 and influenza C virus genomes, we performed phylogenetic analysis. Sequences were aligned using MAFFT[14], and the trees were was inferred using a Maximum Likelihood approach implemented on IQTree[15], with GTR substitution model and 1000 UFBoot replicates. The trees were plotted using the Python package Baltic 0.1.6 (https://github.com/evogytis/baltic). SARS-CoV-2 genomes included in the phylogenetic tree were subsampled from GISAID, whereas the complete HEF gene database from GISAID was used for the influenza virus C phylogeny.

Assessing microbial reads using CZ-ID
FASTQ files from patient NP sample RNASeq data were uploaded to IDseq for analysis using the metagenomics pipeline. Reads per million (rpm) and genome coverage of the alignments for the major bacterial pathobionts detected, *Moraxella catarrhali*s and *Haemophilus influenzae*, are listed in Table S4. Respiratory virus reads were recorded to confirm RVP results or absence of viruses in negative control samples, also shown in Table S4.

Visualization of RNA-Seq data.
Heatmaps: NP sample transcriptomes were visualized using the Qlucore Omics Explorer (v3.7; Qlucore, Lund, Sweden). We created a merged list of DEGs derived from pairwise comparisons between RV, CoV-NL63, or SARS-CoV-2 positive samples and virus-negative controls and performed unsupervised clustering of all samples using this gene list based on RPKM values.

A heatmap was generated using the top 2768 DEG differentially expressed genes, determined by the following cutoffs: $p \leq 0.005$, $q \leq 0.05$. Biological processes for each cluster were identified by Gene ontology (GO) using STRING database version 11.5.

UMAPs: The log of RPKM values from RNASeq was z-score normalized per gene for all genes identified to be differentially expressed. All log operations were base 10 and performed after a pseudocount was added to all zero values which are calculated per feature as one half the maximum observed for that feature unless specified otherwise. These values were then passed to the UMAP function as implemented in the R UMAP package with the n_neighbors parameter set to 5 and default values otherwise to project the data to a 2-dimensional space[16].

Proteomics

Multiplex cytokine measurements were performed in known virus-positive and virus-negative samples and samples discovered in the 2017 screen using the BioPlex 200 HD71 71-plex Human Cytokine Array/Chemokine Array (Eve Technologies, Calgary, AB, Canada). NP swab-associated cell free VTM was shipped overnight on dry ice to Eve Technologies for analysis by BioPlex 200 HD71 multiplex immunoassay. Cytokines that were below the lower limit of quantitation were excluded from downstream analyses.

Visualization of proteomic data.

NP proteome heatmaps were visualized using the Qlucore Omics Explorer (v3.7; Qlucore, Lund, Sweden). SARS-CoV-2 samples were compared using multi-group comparison of three groups: SARS- CoV-2 peak, SARS-CoV-2 end, and negative controls (p-value cutoff <0.05). Virus positive groups (RV, SARS-CoV-2 peak or CoV-NL63) and virus negative samples were compared using multi-group comparisons (p value≤ 0.05 for supervised clustering, Fig 4b, and p value≤ 0.01 for unsupervised clustering, Fig S4). Samples are arranged from low to high viral load based on the sample Ct value.

Electronic medical record data extraction

To evaluate the clinical and demographic data associated with respiratory virus PCR-negative samples screened for SARS-CoV-2 in March 2020, data were extracted from the Yale-New Haven Hospital Observational Medical Outcomes Partnership (OMOP) data repository and analyzed within our computational health platform. All work was done using R (version 3.5.1). Data was extracted from the OMOP data repository using the sparklyr package (version 1.2.0), and the Elixhauser comorbidity data was computed using the comorbidity package (version 0.5.3). Additionally, tables were built using the furniture package (version 1.9.10). The individual Elixhauser categories were combined as follows to yield the Respiratory, Cardiovascular, Diabetes, Cancer, Liver/Kidney, and Other grouping; Respiratory: “chronic pulmonary disease”; Cardiovascular: any of “congestive heart failure”, “cardiac arrhythmias”, “valvular disease”, “pulmonary circulation disorders”, “peripheral vascular disorders”, “hypertension, uncomplicated”, “hypertension, complicated”; Diabetes: “diabetes, uncomplicated” or “diabetes, complicated”; Cancer: any of “lymphoma”, “metastatic cancer”, “solid tumour, without metastasis”; Liver/Kidney: “renal failure” or “liver disease”; Other: any of the remaining categories. The race categories of “Black” and “White” have overlap with the ethnicity category of “Hispanic” in our data, so these were harmonized into non-overlapping groups of “Black, Non-Hispanic”, “White, Non-Hispanic” and “Hispanic”.

Statistical analyses

Correlations between CXCL10 values obtained using Bioplex assay and Ella Simple Plex assay were calculated using GraphPad Prism (v9.3.1, GraphPad Software, San Diego, CA, USA) based on paired measurements of the sample set shown in Fig 4c. Correlation analysis between CXCL10 values measured by ELISA and ELLA was performed using 32 nasopharyngeal swab samples from pediatric patients collected in June 2021 at YNHH, using samples ranging in value from 31 to 470 pg/ml by ELISA. ROC curve for CXCL10 was calculated using IBM SPSS Statistics v 28.0.0.0. For comparisons of Chi-square tests Fisher’s exact test was performed using fisher.test in R with default parameters

Supplementary Figures

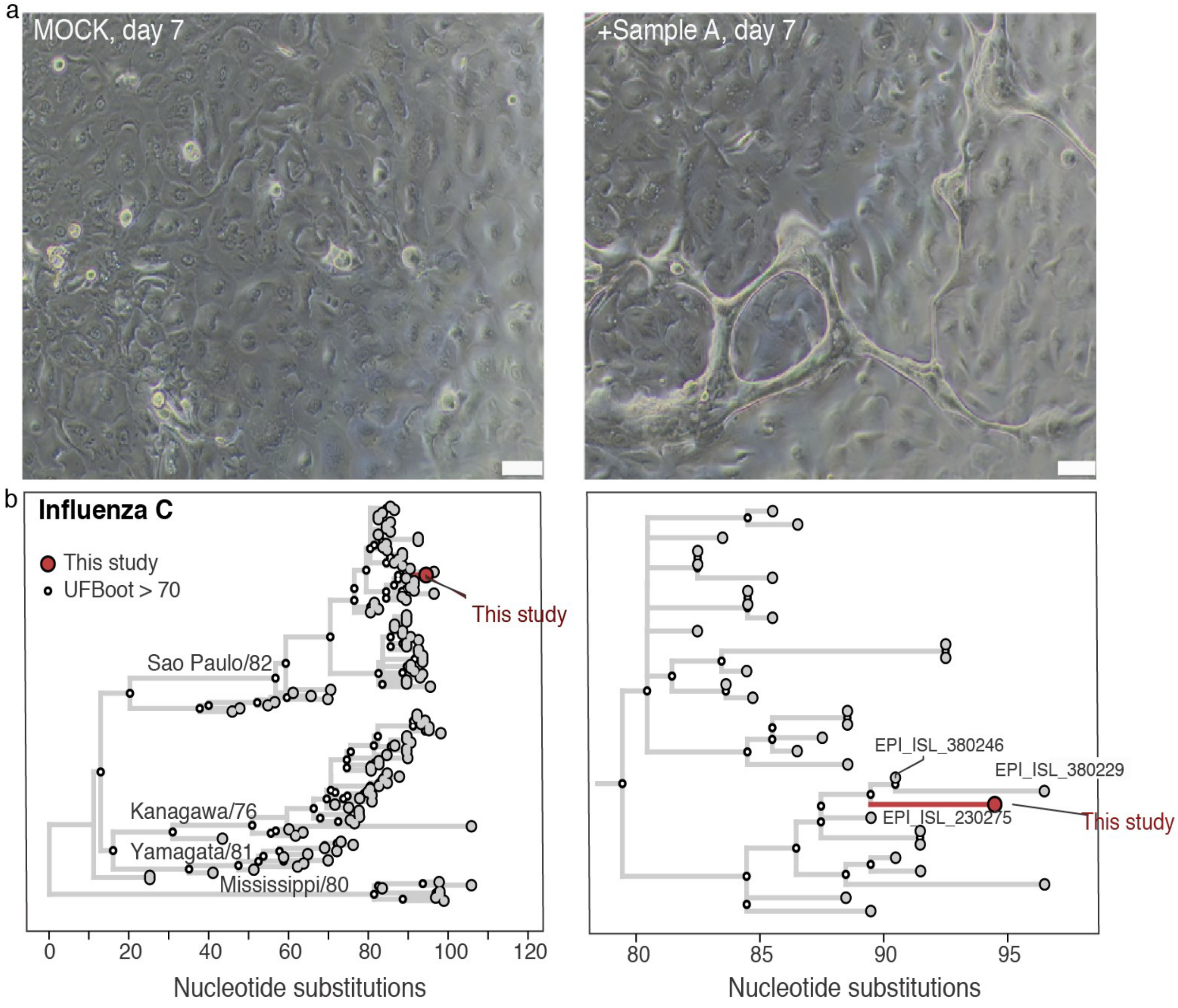

Figure S1 (related to Fig 1). Primary human nasal epithelial cells 7 days post-inoculation with sample A containing influenza C virus and molecular epidemiology of influenza C isolate.

a. Conventionally cultured primary human nasal airway epithelial cells were inoculated with sample A and incubated for 7 days at 37°C. ICV was detected in the supernatant of the culture shown in micrograph B by PCR at day 7. Scale bar = 200 microns. **b**. Maximum likelihood phylogenetic tree of Influenza C viruses, plotted with the python package baltic 0.1.6. The tree was mid-rooted for clarity. The new ICV isolate belongs to the lineage São Paulo/82 (left panel), clustering with viruses from Hong Kong and Japan (right panel).

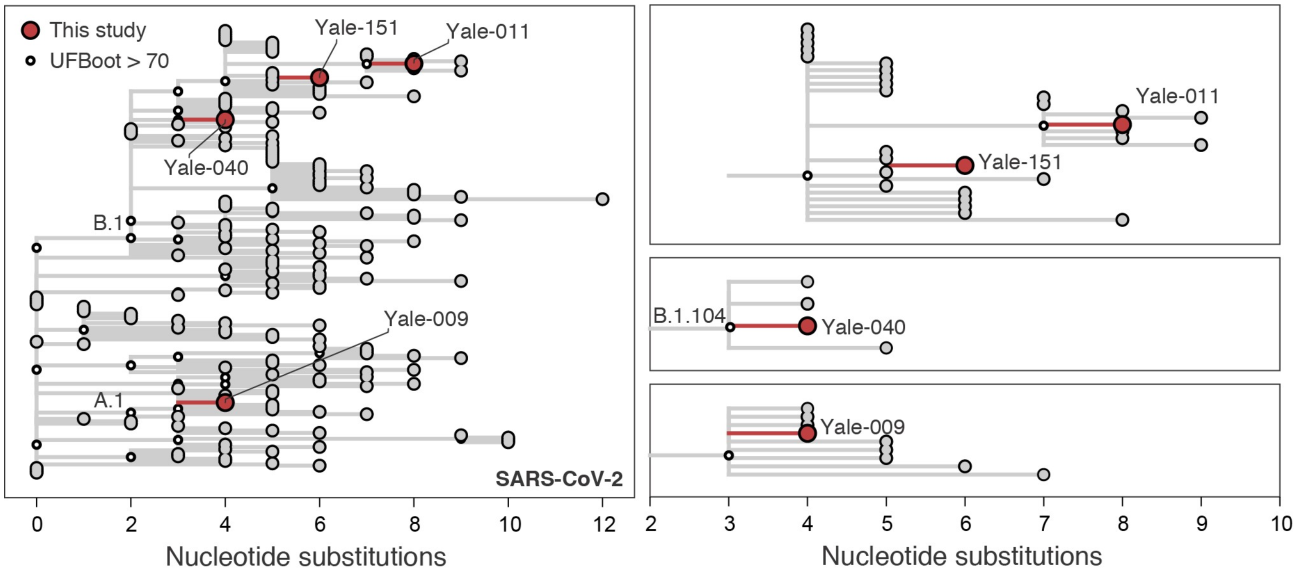

Fig S2. Phylogenetic analysis of four SARS-CoV-2 isolated identified in March 2020 screen. (related to Fig 3) Maximum likelihood phylogenetic tree highlighting the evolutionary history of four SARS-CoV-2 isolates identified in this screen(left panel), and to different lineages and sub-lineages of local and international origins82 (right panel).This tree was rooted at the MRCA of two early isolates from Wuhan: Wuhan/Hu-1/2019 and Wuhan/WH01/2019.The phylogeny was plotted using the python package baltic 0.1.6.

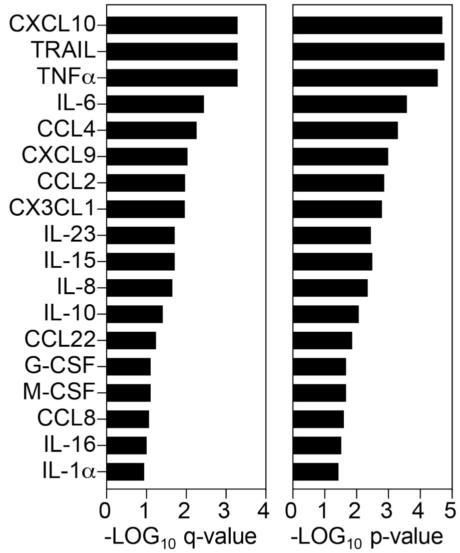

Figure S3 (related to Fig 4). P- and Q- (FDR) values for cytokines differentially elevated in virus positive samples compared to virus negative subjects. Bar graph shows top cytokines ranked based on corresponding q-values (false discovery rate) and p-values from Qlucore analysis of virus positive samples and negative controls included in Fig 4b.

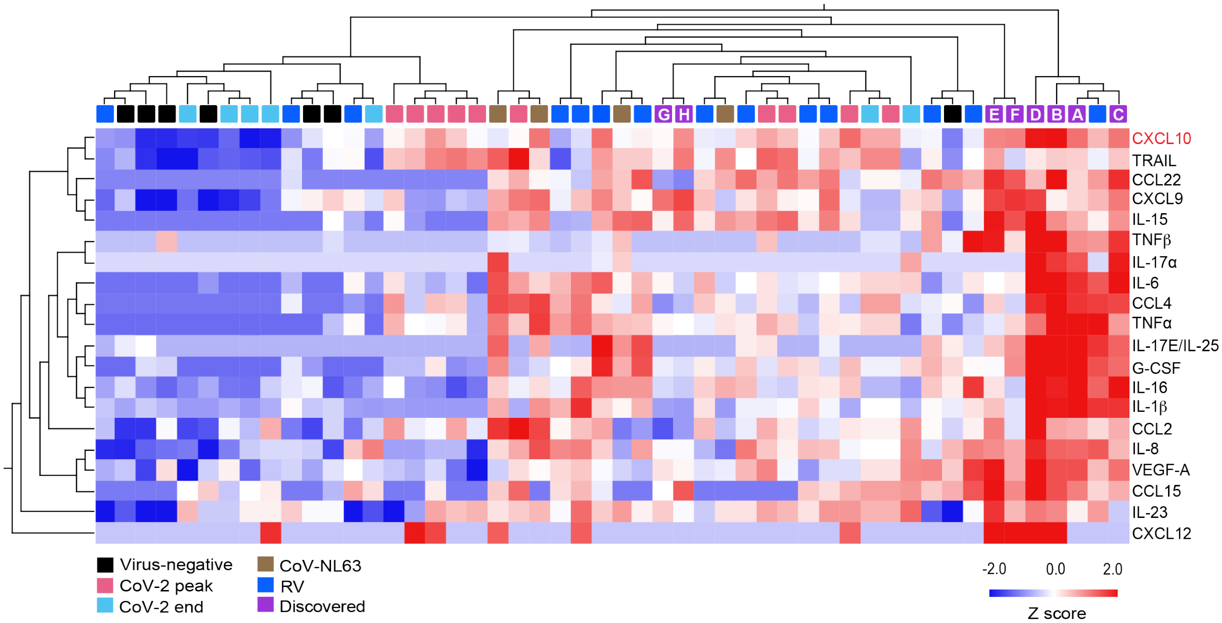

Figure S4 (related to Fig 4). Unsupervised clustering of known virus-positive and virus-negative samples and discovered samples from 2017 screen based on cytokine expression patterns. Heatmap showing top differentially expressed cytokines across samples including virus-negative controls, SARS-CoV-2 peak and end, rhinovirus, CoV-NL63 and samples discovered in the 2017 screen (p≤0.01). Z score represents SD from the mean.

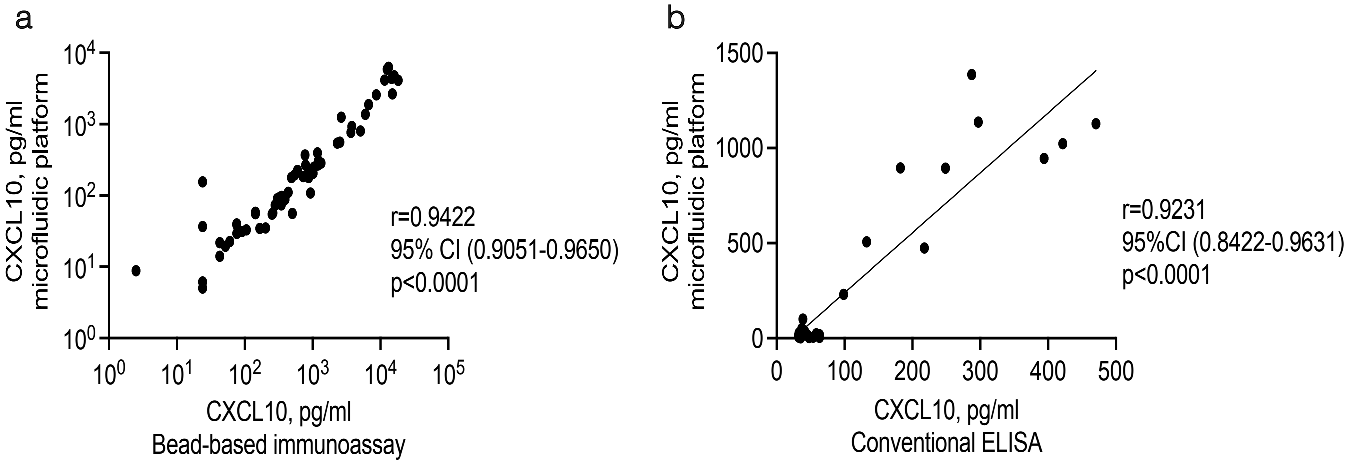

Figure S5 (related to Fig 4). Correlation of CXCL10 values on different assay platforms. a. Correlation of CXCL10 values on bead-based-immunoassay vs. microfluidic assay. **b.** Correlation of CXCL10 values on conventional ELISA vs. microfluidic assay.

Supplementary Tables

Table S1 (related to Fig 1 and 3). Respiratory viruses detected by the Yale-New Haven Hospital PCR panel, 2017 and 2020

Rhinovirus
Influenza A and B (IAV, IBV)
Parainfluenza 1, 2, and 3 (PIV 1-3)
Respiratory syncytial virus A and B (RSV A, B)
Human metapneumovirus (hMPV)
Adenoviruses (AdV)
Parainfluenza 4 (PIV-4)*
Seasonal coronaviruses (CoV-OC43, 229E, NL63, HKU1)*

*tests added in 2019

Table S2. (related to Fig 1) Patient demographics and clinical presentation associated with the 251 nasopharyngeal samples testing negative for respiratory viruses, week 4, January 2017.

Age	N (%)†
<5	9 (3.6)
6-15	2 (0.8)
16-25	13 (5.2)
26-45	28 (11.2)
46-55	35 (13.9)
56-65	56 (22.3)
>65	108 (43.0)
Gender	
Male	98 (39.0)
Female	153 (61.0)
Race/Ethnicity	
White	143 (57.0)
Black	67 (26.7)
Hispanic	32 (12.8)
Other/Unknown	9 (3.6)
Patient Status	
Inpatient	202 (80.5)
Outpatient	23 (9.2)
ED	21 (8.4)
Unknown	5 (2.0)
Presenting symptoms	
Respiratory	134 (53.4)
Fever	49 (19.5)
Cardiac	26 (10.4)
Altered mental state Fatigue	22 (8.8)<br>8 (3.6)
Other	95 (37.8)
Comorbidities	
Respiratory	84 (33.5)
Cardiovascular	67 (26.7)
Diabetes	60 (23.9)
Cancer	51 (20.3)
Liver/kidney disease	48 (19.1)
Other	81 32.3)
Ordering Department	
General medicine	148 (59.0)
ICU/Surgery	35 (13.9)
Oncology	22 (8.8)
ED	20 (8.0)
Outpatient	16 (6.4)
Other/Unknown	10 (4.0)

†*Percentages may not add up to 100% due to multiple symptoms/comorbidities per single patient*

Table S3 (related to Fig 1). Description of viral PCR negative samples from week 4, January 2017

	CXCL10 low n=191 N (%)†	CXCL10 high n=32 N(%)†	
Age			0.038
<5	4 (2.1)	3 (9.4)	
6-15	2 (1.1)	0 (0.0)	
16-25	7 (3.7)	5 (15.6)	
26-45	21 (11.0)	3 (9.4)	
46-55	29 (15.2)	4 (12.5)	
56-65	42 (22.0)	5 (15.6)	
>65	86 (45.0)	12 (37.5)	
Sex			0.67
Male	76 (39.8)	14 (43.8)	
Female	115 (60.2)	18 (56.3)	
Race/Ethnicity			0.16
White	105 (55.0)	24 (75.0)	
Black	52 (27.2)	6 (18.8)	
Hispanic	25 (13.1)	2 (6.3)	
Other/Unknown	9 (4.7)	0 (0.0)	
Patient Status			0.68
Inpatient	157 (82.2)	25 (78.1)	
Outpatient	15 (7.9)	4 (12.5)	
ED	15 (7.9)	3 (9.4)	
Unknown	4 (2.1)	0 (0.0)	
Presenting symptoms*			
Respiratory symptoms	95 (49.7)	20 (62.5)	0.18
Fever	37 (19.4)	6 (18.8)	0.93
Cardiac	20 (10.5)	1 (3.1)	0.32
Altered mental state	16 (8.4)	4 (12.5)	0.50
Fatigue	3 (1.6)	5 (15.6)	<0.001
Other	65 (34.0)	14 (43.8)	0.29
Comorbidities*			
Respiratory	68 (33.6)	10 (31.3)	0.63
Cardiovascular	57 (29.8)	5 (15.6)	0.097
Diabetes	49 (25.7)	5 (15.6)	0.24
Cancer	37 (19.4)	8 (25.0)	0.46
Liver/kidney disease	42 (22.0)	4 (12.5)	0.22
Other	62 (32.5)	9 (28.1)	0.63
Ordering Department			0.92
General medicine	113 (59.2)	17 (53.1)	
ICU/Surgery	27 (14.1)	6 (18.8)	
Oncology	18 (9.4)	2 (6.3)	
ED	15 (7.9)	3 (9.4)	
Other/Unknown	7 (3.7)	2 (6.3)	
Outpatient	11 (5.8)	2 (6.3)	

Comparisons were performed using a global X^2 test or individual X^2 test if indicated with asterisk (*)
Threshold for significance $p<0.003$ to adjust for multiple comparisons using the Bonferroni correction
(†) *Some percentages may not add up to 100% due to multiple symptoms/comorbidities per single patient*

Table S4. Samples used for transcriptomics (n=53)

Negative Controls	Bacterial pathobiont	reads per million	% genome coverage	Depth of coverage	Virus-top hit	reads per million	% genome coverage	Depth of coverage	Ct value	Age	Sex	Clinical History
	--				none					60-70	M	
	--				none					60-70	F	
	--				none					60-70	F	
	--				none					60-70	F	
	Moraxella catarrhalis	3.62E+05	0.5	40.3	none					60-70	F	
	--				none					60-70	F	
	*				none					70-80	M	
	--				none					60-70	F	

Rhinovirus positive	Bacterial pathobiont	reads per million	% genome coverage	Depth of coverage	Virus-top hit	reads per million	% genome coverage	Depth of coverage	Ct value	Age	Sex	Clinical History
	Moraxella catarrhalis	5.0E+05	60.9	24.8	Rhinovirus C	11.2	45.2	0.88	29.5	0-5	M	ARI, immunosupressed
	Moraxella catarrhalis	5.1E+05	83.9	30.3	Rhinovirus C	3.6	13.3	0.26	30.3	40-50	F	ARI, immunosuppressed
	Haemophilus influenzae	5.1E+05	27.3	4.7	Rhinovirus C	61.1	99.5	4.9	26.1	0-5	F	ARI, outpatient
	Moraxella catarrhalis	2.0E+05	63.8	11.7								
	Haemophilus influenzae	7.4E+05	95.6	59.3	Rhinovirus A	299.9	99.8	54.2	24	0-5	F	ARI, outpatient
					HRSV/B/Buenos Aires 2016	911.2	100	78.9				
					Human coronavirus NL63	118.4	88	5.3				
	Haemophilus influenzae	1.0E+05	1.7	12.5	Rhinovirus A	2541.7	99.4	244.3	22.1	90-100	F	Pneumonia
	--				Rhinovirus A	1131	99.3	146.5	24.5	60-70	F	ARI
	--				Rhinovirus C	210713.8	97.1	6702.4	19	20-30	F	Exacerbation of chronic lung disease
	--				Rhinovirus C	3290.1	100	612.1	22.9	70-80	M	ARI
	--				Rhinovirus A	1766	99.7	1064	22.4	20-30	F	Asthma exacerbation
	--				Rhinovirus C	2358.9	99.8	619	26.2	29	F	ARI
	--				Rhinovirus C	67.2	99.6	32.6	27.8	44	F	ARI

**Seasonal CoV-NL63**	**Bacterial pathobiont**	**reads per million**	**% genome coverage**	**Depth of coverage**	**Virus-top hit**	**reads per million**	**% genome coverage**	**Depth of coverage**	**Ct value**	**Age**	**Sex**	**Clinical History**
	--				Human coronavirus NL63	502363.8	100	2338.7	13.4	50-60	M	Fever, cancer
	--				Human coronavirus NL63	107740.8	100	6009.2	13.8	50-60	M	Cough, immunospressed
	--				Human coronavirus NL63	252.4	35.1	7.8	19.6	11	M	ARI, dehydration
	--				Human coronavirus NL63	2743.3	100	398.3	14	4	M	ARI, cancer
**SARS CoV-2**	**Bacterial pathobiont**	**reads per million**	**% genome coverage**	**Depth of coverage**	**Virus-top hit**	**reads per million**	**% genome coverage**	**Depth of coverage**	**Ct value**	**Age**	**Sex**	**Inpatient vs. Outpatient**
	Moraxella catarrhalis	1.8E+05	0.1	10.3	Severe acute respiratory syndrome-related coronavirus	51372.5	95.4	2600.8	17.5	40-50	F	Inpatient
	*				Severe acute respiratory syndrome-related coronavirus	303.1	85.4	11.7	20.5	20-30	F	Outpatient
	*				Severe acute respiratory syndrome-related coronavirus	9.3	5.9	0.14	26.8	20-30	M	Inpatient
	*				Severe acute respiratory syndrome-related coronavirus	73324.0	99.8	1274.3	13	50-60	M	Outpatient
	*				Severe acute respiratory syndrome-related coronavirus	6327.9	99.9	286.9	17.7	40-50	M	Outpatient
	*				Severe acute respiratory syndrome-related coronavirus	23881.1	99.8	520.8	15.3	20-30	M	Outpatient
	Moraxella catarrhalis	1.9E+05	0.5	14.7	Severe acute respiratory syndrome-related coronavirus	10537.2	100	785	18.3	70-80	M	Outpatient
	--				Severe acute respiratory syndrome-related coronavirus	92039.3	99.4	5595	NA	60-70	M	Inpatient
	--				Severe acute respiratory syndrome-related coronavirus	194981.7	76.7	4277.8	12.3	80-90	F	Inpatient
	--				Severe acute respiratory syndrome-related coronavirus	340.8	81.6	8.1	24.7	60-70	M	Inpatient
	--				Rhinovirus C	17.4	99.8	24.5				
	--				Severe acute respiratory syndrome-related coronavirus	6890.7	99.8	814	20.7	90-100	F	Inpatient
	--				Severe acute respiratory syndrome-related coronavirus	220686.9	99.8	5578.8	17.2	70-80	M	Inpatient
	--				Severe acute respiratory syndrome-related coronavirus	2.5	NA	NA	31.6	30-40	M	Inpatient
	--				Severe acute respiratory syndrome-related coronavirus	9426.1	99.8	477.5	18.1	80-90	M	Inpatient
	--				Severe acute respiratory syndrome-related coronavirus	11.9	3.5	0.11	32	50-60	F	Inpatient
	--				Severe acute respiratory syndrome-related coronavirus	6572.3	99.9	531.2	20.8	70-80	F	Inpatient
	Moraxella catarrhalis	3.2E+04	2.4	4.7	Severe acute respiratory syndrome-related coronavirus	14547.2	99.8	658.7	17.9	90-100	F	Inpatient
	--				Severe acute respiratory syndrome-related coronavirus	17409.6	99.9	531.3	20.8	30-40	M	Inpatient
	Moraxella catarrhalis	4.5E+04	1.3	10.7	Severe acute respiratory syndrome-related coronavirus	2083.5	99.8	77.4	23	60-70	M	Inpatient
	--				Severe acute respiratory syndrome-related coronavirus	160815.0	70.2	282.8		60-70	M	Inpatient

	*				Severe acute respiratory syndrome-related coronavirus	39837.7	99.9	1239	16.1	30-40	M	Outpatient
	--				Severe acute respiratory syndrome-related coronavirus	6847.7	99.8	205.3	23.2	30-40	M	Inpatient
	*				Severe acute respiratory syndrome-related coronavirus	5389.2	99	170.3	17.9	60-70	F	Outpatient
	*				Severe acute respiratory syndrome-related coronavirus	12608.3	99.8	673.2	22.7	60-70	M	Inpatient
	--				Severe acute respiratory syndrome-related coronavirus	164033.8	98.3	5246.1	14.5	60-70	M	Inpatient
	--				Severe acute respiratory syndrome-related coronavirus	19613.1	99.8	450.2	19.3	60-70	M	Inpatient
	--				Severe acute respiratory syndrome-related coronavirus	1873.0	99.7	319.8	19.7	70-80	M	Inpatient
	--				Severe acute respiratory syndrome-related coronavirus	61489.0	97.7	2762.2	16.2	70-80	M	Inpatient
	Moraxella catarrhalis	7.7E+04	7.6	6.2	Severe acute respiratory syndrome-related coronavirus	69987.1	96	3983.2	14.6	70-80	F	Inpatient
	Moraxella catarrhalis	1.5E+04	0.2	12.1	Severe acute respiratory syndrome-related coronavirus	12.6	38.1	1.7	28.7	90-100	F	Inpatient

**significant reads from H. parainfluenza*

Table S5. Top 20 GO biological process for each cluster shown in Figure 2 (STRING v11.5)

Cluster	#GO term ID	Term description	Observed gene count	Background gene count	Strength	False discovery rate
1	GO:0006413	translational initiation	18	141	1.09	9.80E-10
1	GO:0000184	nuclear-transcribed mRNA catabolic process, nonsense-mediated decay	16	119	1.11	4.76E-09
1	GO:0006614	SRP-dependent cotranslational protein targeting to membrane	15	96	1.18	4.76E-09
1	GO:0019080	viral gene expression	16	132	1.07	1.06E-08
1	GO:0019083	viral transcription	15	115	1.1	1.27E-08
1	GO:0006612	protein targeting to membrane	17	165	0.99	1.31E-08
1	GO:0044270	cellular nitrogen compound catabolic process	21	422	0.68	9.24E-06
1	GO:0034655	nucleobase-containing compound catabolic process	19	373	0.69	2.93E-05
1	GO:0046700	heterocycle catabolic process	20	422	0.66	3.65E-05
1	GO:0019439	aromatic compound catabolic process	20	437	0.64	5.95E-05
1	GO:0006605	protein targeting	18	356	0.69	6.49E-05
1	GO:1901361	organic cyclic compound catabolic process	20	472	0.61	0.00017
1	GO:0065007	biological regulation	159	12171	0.1	0.00094
1	GO:0002181	cytoplasmic translation	8	72	1.03	0.0013
1	GO:0050789	regulation of biological process	152	11475	0.1	0.0013
1	GO:0072594	establishment of protein localization to organelle	17	433	0.58	0.0031
1	GO:0072657	protein localization to membrane	18	495	0.54	0.0043
1	GO:0016032	viral process	23	776	0.45	0.0055
1	GO:0040012	regulation of locomotion	26	969	0.41	0.0072
1	GO:0044403	symbiotic process	24	865	0.43	0.0091
2	GO:0044782	cilium organization	74	360	0.69	3.63E-22
2	GO:0060271	cilium assembly	67	339	0.67	3.19E-19
2	GO:0120031	plasma membrane bounded cell projection assembly	68	433	0.57	6.06E-15
2	GO:0030031	cell projection assembly	69	450	0.56	7.71E-15
2	GO:0007017	microtubule-based process	90	727	0.47	9.08E-15
2	GO:0007018	microtubule-based movement	56	334	0.6	3.11E-13
2	GO:0030030	cell projection organization	107	1170	0.34	7.40E-10
2	GO:0042073	intraciliary transport	21	52	0.98	1.29E-09
2	GO:0099111	microtubule-based transport	35	185	0.65	6.42E-09
2	GO:0120036	plasma membrane bounded cell projection organization	101	1122	0.33	6.42E-09
2	GO:0098840	protein transport along microtubule	22	67	0.89	7.70E-09
2	GO:0070925	organelle assembly	75	735	0.39	1.96E-08
2	GO:0035735	intraciliary transport involved in cilium assembly	17	40	1.01	6.29E-08
2	GO:0001578	microtubule bundle formation	23	101	0.73	9.26E-07
2	GO:0010970	transport along microtubule	28	155	0.63	1.33E-06
2	GO:0035082	axoneme assembly	19	70	0.81	2.08E-06
2	GO:0003341	cilium movement	24	130	0.64	1.24E-05
2	GO:0030705	cytoskeleton-dependent intracellular transport	29	195	0.55	2.78E-05
2	GO:0000226	microtubule cytoskeleton organization	50	492	0.38	4.07E-05

2	GO:0006928	movement of cell or subcellular component	104	1501	0.22	0.00068
3	GO:0002376	immune system process	405	2481	0.41	1.81E-64
3	GO:0006955	immune response	300	1588	0.47	1.35E-56
3	GO:0001775	cell activation	232	1075	0.53	1.12E-50
3	GO:0045321	leukocyte activation	208	929	0.55	4.70E-47
3	GO:0002252	immune effector process	205	969	0.52	4.57E-43
3	GO:0002274	myeloid leukocyte activation	157	585	0.63	1.04E-42
3	GO:0048583	regulation of response to stimulus	488	4114	0.27	4.09E-42
3	GO:0002366	leukocyte activation involved in immune response	159	626	0.6	7.35E-41
3	GO:0043299	leukocyte degranulation	142	506	0.65	2.45E-40
3	GO:0002275	myeloid cell activation involved in immune response	144	522	0.64	2.88E-40
3	GO:0036230	granulocyte activation	141	502	0.65	3.83E-40
3	GO:0042119	neutrophil activation	140	497	0.65	5.47E-40
3	GO:0002444	myeloid leukocyte mediated immunity	142	516	0.64	1.16E-39
3	GO:0016192	vesicle-mediated transport	282	1805	0.39	5.04E-39
3	GO:0002283	neutrophil activation involved in immune response	137	488	0.65	5.20E-39
3	GO:0045055	regulated exocytosis	163	697	0.57	2.06E-38
3	GO:0043312	neutrophil degranulation	135	484	0.64	3.47E-38
3	GO:0002446	neutrophil mediated immunity	136	495	0.64	6.57E-38
3	GO:0050896	response to stimulus	750	8046	0.17	1.77E-37
3	GO:0002443	leukocyte mediated immunity	153	641	0.58	8.03E-37
4	GO:0006955	immune response	121	1588	0.77	4.93E-57
4	GO:0002376	immune system process	141	2481	0.64	1.78E-54
4	GO:0006952	defense response	107	1296	0.81	2.03E-52
4	GO:0051707	response to other organism	103	1256	0.8	7.20E-50
4	GO:0098542	defense response to other organism	87	900	0.87	3.47E-46
4	GO:0044419	interspecies interaction between organisms	116	1899	0.67	2.21E-45
4	GO:0051607	defense response to virus	50	210	1.27	3.24E-41
4	GO:0009615	response to virus	55	293	1.16	6.47E-41
4	GO:0045087	innate immune response	73	703	0.9	9.29E-40
4	GO:0009605	response to external stimulus	113	2310	0.58	5.21E-35
4	GO:0002682	regulation of immune system process	91	1514	0.67	4.72E-33
4	GO:0002252	immune effector process	72	969	0.76	2.40E-30
4	GO:0019221	cytokine-mediated signaling pathway	62	678	0.85	3.37E-30
4	GO:0034097	response to cytokine	75	1101	0.72	1.53E-29
4	GO:0006950	response to stress	129	3485	0.46	1.82E-29
4	GO:0050776	regulation of immune response	67	896	0.76	3.75E-28
4	GO:0071345	cellular response to cytokine stimulus	70	1013	0.73	1.17E-27
4	GO:0002684	positive regulation of immune system process	68	949	0.74	1.22E-27
4	GO:0034340	response to type I interferon	26	72	1.45	2.20E-24
4	GO:0060337	type I interferon signaling pathway	25	67	1.46	1.11E-23

Table S6 (related to Fig 3). Comparison of patient features and CXCL10 levels for respiratory virus panel-negative samples testing SARS-CoV-2-negative or positive, March 3-14, 2020.

	Total n=375 N (%)	SARS-CoV-2- n=371 N (%)	SARS-CoV-2+ n=4 N (%)	Fishers exact test p value
Gender				0.3638
Female	191 (50.9)	190 (51.2)	1 (25)	
Male	184 (49.1)	181 (48.8)	3 (75)	
Race, ethnicity				0.6159
Black, Non-Hispanic	70 (18.7)	70 (18.9)	0 (0)	
White, Non-Hispanic	244 (65.1)	241 (65)	3 (75)	
Hispanic	45 (12)	44 (11.9)	1 (25)	
Other	16 (4.3)	16 (4.3)	0 (0)	
Age				0.1952
0-5 years	9 (2.4)	8 (2.2)	1 (25)	
5-16 years	10 (2.7)	10 (2.7)	0 (0)	
16-25 years	11 (2.9)	11 (3)	0 (0)	
25-45 years	41 (10.9)	41 (11.1)	0 (0)	
45-65 years	117 (31.2)	115 (31)	2 (50)	
> 65 years	187 (49.9)	186 (50.1)	1 (25)	
CXCL10 level*				4.4E-05
<100pg/ml	343 (91.5)	343 (92.5)	0 (0)	
>100pg/ml	32 (8.5)	28 (7.5)	4 (100)	
Status				0.08859
Inpatient	298 (79.5)	296 (79.8)	2 (50)	
ED	44 (11.7)	43 (11.6)	1 (25)	
Outpatient	13 (3.5)	12 (3.2)	1 (25)	
Other	20 (5.3)	20 (5.4)	0 (0)	
Respiratory				0.1254
Negative	198 (52.8)	194 (52.3)	4 (100)	
Positive	177 (47.2)	177 (47.7)	0 (0)	
Cardiovascular				0.03658
Negative	84 (22.4)	81 (21.8)	3 (75)	
Positive	291 (77.6)	290 (78.2)	1 (25)	
Diabetes				0.3076
Negative	252 (67.2)	248 (66.8)	4 (100)	
Positive	123 (32.8)	123 (33.2)	0 (0)	
Cancer				0.5796
Negative	292 (77.9)	288 (77.6)	4 (100)	
Positive	83 (22.1)	83 (22.4)	0 (0)	
Liver / Kidney				1
Negative	228 (60.8)	225 (60.6)	3 (75)	
Positive	147 (39.2)	146 (39.4)	1 (25)	
Other				0.04585
Negative	91 (24.3)	88 (23.7)	3 (75)	
Positive	284 (75.7)	283 (76.3)	1 (25)	

**Significant difference between SARS-CoV-2 positive and SARS-CoV-2 negative*

Threshold for significance p<0.005 to adjust for multiple comparisons using the Bonferroni correction

Table S7 (related to Fig 3). Summary statistics for MinION sequencing of SARS-CoV-2 positive samples.

Sample ID	Sample type	CT Value	Reads to Barcode	Reads to SARS-CoV-2 Genome	Reads Aligned	Genome Coverage	Avg DOC
Yale-009	NP	21	123,547	123,543	100	97.4	200+
Yale-011	NP	28	159,675	156,799	98.2	96.8	200+
Yale-040	NP	22	345,561	342,796	99.2	98.6	200+
Yale-151	NP	15	243,524	239,856	98.4	99.6	200+

NP= nasopharyngeal swab

DOC=Depth of coverage in reads across SARS-CoV-2 genome

Table S8. Samples used for proteomics analysis, n=50 (Fig 4)

Category	Virus positive?	Hflu/Mcat by RNASeq?	Age	Sex	Ct value, viral PCR
Virus-negative, adult	NO	NO (<10,000 RPM)	60s	M	-
Virus-negative, adult	NO	NO	60s	F	-
Virus-negative, adult	NO	NO	60s	F	-
Virus-negative, adult	NO	NO	70s	M	-
Virus-negative, adult	NO	Detected (>10,000 RPM)	60s	F	-
Virus-negative, adult	NO	NO	60s	F	-
Virus-negative, adult	NO	NO	60s	F	-
CoV-NL63	YES	NO	50s	M	13.4
CoV-NL63	YES	NO	50s	M	13.8
CoV-NL63	YES	NO	10-15yrs	M	19.6
CoV-NL63	YES	NO	60s	F	14
Rhinovirus	YES	NO	30s	F	27.7
Rhinovirus	YES	NO	<5 yrs	M	30.4
Rhinovirus	YES	NO	60s	F	34.5
Rhinovirus	YES	NO	77	F	21.8
Rhinovirus	YES	NO	76	M	18.4
Rhinovirus	YES	NO	70s	M	22.9
Rhinovirus	YES	NO	60s	F	24.5
Rhinovirus	YES	NO	20s	F	26.2
Rhinovirus	YES	NO	40s	F	27.8
Rhinovirus	YES	High (>100,000 RPM)	90s	F	22.1
Rhinovirus	YES	High	<5	F	24
Rhinovirus	YES	High	<5yrs	F	26.1
Rhinovirus	YES	High	40s	F	30.3
Rhinovirus	YES	High	<5yrs	M	29.5
Discovered -A	YES (ICV)	High	<5yrs	M	-
Discovered -B	no info	High	<5yrs	F	-
Discovered -C	no info	Detected	50s	M	-
Discovered -D	no info	NO	40s	M	-
Discovered -E	no info	NO	60s	F	-
Discovered -F	no info	Detected	70s	F	-
Discovered -G	no info	NO	20s	M	-
Discovered -H	no info	NO	20s	F	-
COVID, peak viral load	YES	*not done*	70s	F	15.9
COVID, peak viral load	YES	*not done*	80s	F	11.6
COVID, peak viral load	YES	*not done*	90s	F	15.5
COVID, peak viral load	YES	*not done*	60s	M	15.1
COVID, peak viral load	YES	*not done*	80s	M	12.8
COVID, peak viral load	YES	*not done*	50s	M	17.2
COVID, peak viral load	YES	*not done*	70s	F	15.5
COVID, peak viral load	YES	*not done*	90s	F	12.6
COVID, peak viral load	YES	*not done*	50s	M	17.1
COVID, peak viral load	YES	*not done*	70s	F	14.6
COVID, end of disease course	YES	*not done*	60s	M	37.2
COVID, end of disease course	YES	*not done*	80s	M	35.1
COVID, end of disease course	YES	*not done*	50s	M	36.9
COVID, end of disease course	YES	*not done*	70s	F	36.7
COVID, end of disease course	YES	*not done*	90s	F	31.2
COVID, end of disease course	YES	*not done*	50s	M	35.5
COVID, end of disease course	YES	*not done*	70s	F	35.4

REFERENCES CITED

1 Landry, M. L. & Foxman, E. F. Antiviral Response in the Nasopharynx Identifies Patients With Respiratory Virus Infection. *The Journal of infectious diseases* **217**, 897-905, doi:10.1093/infdis/jix648 (2018).

2 Lu, X. *et al.* Rhinovirus Viremia in Patients Hospitalized With Community-Acquired Pneumonia. *The Journal of infectious diseases* **216**, 1104-1111, doi:10.1093/infdis/jix455 (2017).

3 Pierce, V. M., Elkan, M., Leet, M., McGowan, K. L. & Hodinka, R. L. Comparison of the Idaho Technology FilmArray system to real-time PCR for detection of respiratory pathogens in children. *J Clin Microbiol* **50**, 364-371, doi:10.1128/JCM.05996-11 (2012).

4 CDC. CDC 2019-Novel Coronavirus (2019-nCoV) Real-Time RT-PCR Diagnostic Panel (2020). <https://www.fda.gov/media/134922/download>.

5 Aldo, P., Marusov, G., Svancara, D., David, J. & Mor, G. Simple Plex() : A Novel Multi-Analyte, Automated Microfluidic Immunoassay Platform for the Detection of Human and Mouse Cytokines and Chemokines. *Am J Reprod Immunol* **75**, 678-693, doi:10.1111/aji.12512 (2016).

6 Kim, D., Paggi, J. M., Park, C., Bennett, C. & Salzberg, S. L. Graph-based genome alignment and genotyping with HISAT2 and HISAT-genotype. *Nat Biotechnol* **37**, 907-915, doi:10.1038/s41587-019-0201-4 (2019).

7 Pertea, M. *et al.* StringTie enables improved reconstruction of a transcriptome from RNA-seq reads. *Nat Biotechnol* **33**, 290-295, doi:10.1038/nbt.3122 (2015).

8 Love, M. I., Huber, W. & Anders, S. Moderated estimation of fold change and dispersion for RNA-seq data with DESeq2. *Genome Biol* **15**, 550, doi:10.1186/s13059-014-0550-8 (2014).

9 Kim, D., Paggi, J. M., Park, C., Bennett, C. & Salzberg, S. L. Graph-based genome alignment and genotyping with HISAT2 and HISAT-genotype. *Nature biotechnology* **37**, 907-915, doi:10.1038/s41587-019-0201-4 (2019).

10 Vogels, C. B. F. *et al.* Analytical sensitivity and efficiency comparisons of SARS-CoV-2 RT-qPCR primer-probe sets. *Nat Microbiol* **5**, 1299-1305, doi:10.1038/s41564-020-0761-6 (2020).

11 Fauver, J. R. *et al.* Coast-to-Coast Spread of SARS-CoV-2 during the Early Epidemic in the United States. *Cell*, doi:10.1016/j.cell.2020.04.021 (2020).

12 Quick, J. *et al.* Multiplex PCR method for MinION and Illumina sequencing of Zika and other virus genomes directly from clinical samples. *Nat Protoc* **12**, 1261-1276, doi:10.1038/nprot.2017.066 (2017).

13 Loman, N. J., Quick, J. & Simpson, J. T. A complete bacterial genome assembled de novo using only nanopore sequencing data. *Nat Methods* **12**, 733-735, doi:10.1038/nmeth.3444 (2015).

14 Katoh, K. & Standley, D. M. MAFFT multiple sequence alignment software version 7: improvements in performance and usability. *Mol Biol Evol* **30**, 772-780, doi:10.1093/molbev/mst010 (2013).

15 Minh, B. Q. *et al.* IQ-TREE 2: New Models and Efficient Methods for Phylogenetic Inference in the Genomic Era. *Mol Biol Evol* **37**, 1530-1534, doi:10.1093/molbev/msaa015 (2020).

16 L. McInnes, J. H., and J. Melville UMAP: Uniform Manifold Approximation and Projection for Dimension Reduction, v3. *arXiv.org*, doi:https://arxiv.org/abs/1802.03426 (2018).
